# Abundant neuroprotective chaperone Lipocalin-type prostaglandin D synthase (L-PGDS) disassembles the Amyloid-β fibrils

**DOI:** 10.1038/s41598-019-48819-5

**Published:** 2019-08-29

**Authors:** Bhuvaneswari Kannaian, Bhargy Sharma, Margaret Phillips, Anup Chowdhury, Malathy S. S. Manimekalai, Sunil S. Adav, Justin T. Y. Ng, Ambrish Kumar, Sierin Lim, Yuguang Mu, Siu K. Sze, Gerhard Grüber, Konstantin Pervushin

**Affiliations:** 1 School of Biological Sciences, Nanyang Technological University, Singapore, 637551 Singapore; 2 Singapore Phenome Centre, Lee Kong Chian School of Medicine, Nanyang Technological University, Singapore, 636921 Singapore; 3 School of Chemical and Biomedical Engineering, Nanyang Technological University, Singapore, 637459 Singapore

**Keywords:** Chaperones, Structural biology

## Abstract

Misfolding of Amyloid β (Aβ) peptides leads to the formation of extracellular amyloid plaques. Molecular chaperones can facilitate the refolding or degradation of such misfolded proteins. Here, for the first time, we report the unique ability of Lipocalin-type Prostaglandin D synthase (L-PGDS) protein to act as a disaggregase on the pre-formed fibrils of Aβ(1–40), abbreviated as Aβ40, and Aβ(25–35) peptides, in addition to inhibiting the aggregation of Aβ monomers. Furthermore, our proteomics results indicate that L-PGDS can facilitate extraction of several other proteins from the insoluble aggregates extracted from the brain of an Alzheimer’s disease patient. In this study, we have established the mode of binding of L-PGDS with monomeric and fibrillar Aβ using Nuclear Magnetic Resonance (NMR) Spectroscopy, Small Angle X-ray Scattering (SAXS), and Transmission Electron Microscopy (TEM). Our results confirm a direct interaction between L-PGDS and monomeric Aβ40 and Aβ(25–35), thereby inhibiting their spontaneous aggregation. The monomeric unstructured Aβ40 binds to L-PGDS via its C-terminus, while the N-terminus remains free which is observed as a new domain in the L-PGDS-Aβ40 complex model.

## Introduction

Alzheimer’s disease (AD) is a devastating neurodegenerative condition which is projected to be a significant risk factor for the global population by the year 2050, affecting more than 13.8 million people in the United States alone^1^. The ‘amyloid cascade hypothesis,’ one of the critical theories on AD pathology, postulates that polymerization of Amyloid β (Aβ) in the brain is a major pathological event in AD^2,3^. Soluble Aβ in the form of small oligomers, Aβ-derived diffusible ligands, and protofibrils are among the major toxic contributors towards AD pathology^4^. In neuroprotection, the molecular chaperones play an important role by inhibiting protein aggregation in the brain^5,6^. Simultaneously, disaggregation of the pre-formed insoluble aggregates is also crucial to mediate therapeutic response to AD pathology^7^. In this context, Aβ aggregation and accumulation is also a consequence of the deficiency in Aβ specific chaperones which can confer the neuroprotective function^8,9^.

L-PGDS, also known as β-trace protein, is proposed to be a major endogenous Aβ chaperone capable of inhibiting Aβ40 and Aβ42 aggregation^10^. It is a major brain-derived protein abundant in the human cerebrospinal fluid (CSF), second only to Albumin^11,12^. L-PGDS is involved in the regulation of various neurological processes, and its altered expression levels in the brain lead to many disease conditions^13,14^. Endogenous L-PGDS localizes with amyloid plaques in both Tg2576 AD mice and patient brain and inhibits Aβ aggregation^10^. L-PGDS also plays a protective role after an ischemic stroke, promotes chemotactic migration of microglial cells and recruitment of astrocytes at the site of injury and can scavenge the reactive oxygen species (ROS) through the thiol group of Cysteine residue^15–17^. The expression of the *ptgds* gene is upregulated in AD phenotypes and positively correlated with amyloid plaques^18,19^. Its expression in the prefrontal cortex of the human brain is associated with clinical and pathological traits of AD, where its level of expression is higher than in cases of other amyloidogenic diseases like Amyotrophic Lateral Sclerosis (ALS) and Parkinson’s Disease (PD)^19,20^. The mechanism of the chaperone activity of L-PGDS has been examined in this study. Here, we show that in addition to its protective role as a chaperone, L-PGDS is also a unique extracellular disaggregase capable of breaking down pre-formed Aβ fibrils *in vitro*.

Intracellular chaperone machinery mainly comprises heat shock proteins (Hsp) which work together as complexes to prevent the aggregation of misfolded amyloids^21^. Degradation of amyloids involves the transport of unfolded proteins to lysosomes or proteasomes by these chaperones^7^. Proteasomes such as ubiquitin-specific protease (USP14) and 26S assist in the degradation of amyloids^22,23^. Besides, extracellular chaperones like clusterin, haptoglobin, and α2 macroglobulin can also bind to misfolded proteins and transport them to the scavenger receptors promoting their degradation^5,24^. Hsp104 in coordination with Hsp70 and its co-chaperone complex with Hsp40, as well as Hsp110, utilize energy from the hydrolysis of ATP for disaggregation of aggregated prion fibrils in yeast systems^25,26^.

Similarly, the same co-chaperone complex sans Hsp104 affects the solubilization of amyloid aggregates in metazoa^27,28^. The general mechanism for amyloid disaggregation involves the disruption of non-covalent interactions or requires energy attained through ATP hydrolysis. For therapeutic purposes, antibodies are the most tested candidates for their potential as protein disaggregases to target the misfolded amyloids and degrade them into smaller units which can be sequestered out of the body^29^. Monoclonal antibodies can deteriorate the morphology of Aβ aggregates and disrupt their fibrillar structure. Unfortunately, one such antibody, Aducanumab failed to treat AD in the clinical trial recently^30^. Here, we report that L-PGDS forms a 1:1 complex with Aβ40 peptide, while its binding site and mode of interaction with monomeric Aβ40 peptide being different from its catalytic site^31^. Aβ(25–35), a small fragment of Aβ(1–42), a fast aggregating peptide which retains its neurotoxic properties, has shown increased oxidative stress and neuronal damage in rats^32,33^. We show that L-PGDS delays the primary and secondary nucleation of Aβ40 monomers upon binding and prevents the aggregation of Aβ40 and Aβ(25–35) peptides. TEM images show the localization of L-PGDS at the growing tips of the preformed Aβ fibrils followed by their disruption. Our NMR studies together with Molecular Dynamics (MD) modeling provide a structural model of L-PGDS in complex with Aβ40 highlighting the binding interface and confirming its interaction with the residues 25–35 of the amyloid peptide. In this study, we have demonstrated the unexplored function of L-PGDS as a potential Aβ disaggregase and further reinforced its role as a major amyloid-β chaperone.

## Results

### L-PGDS is a disaggregase

**Figure 1:**
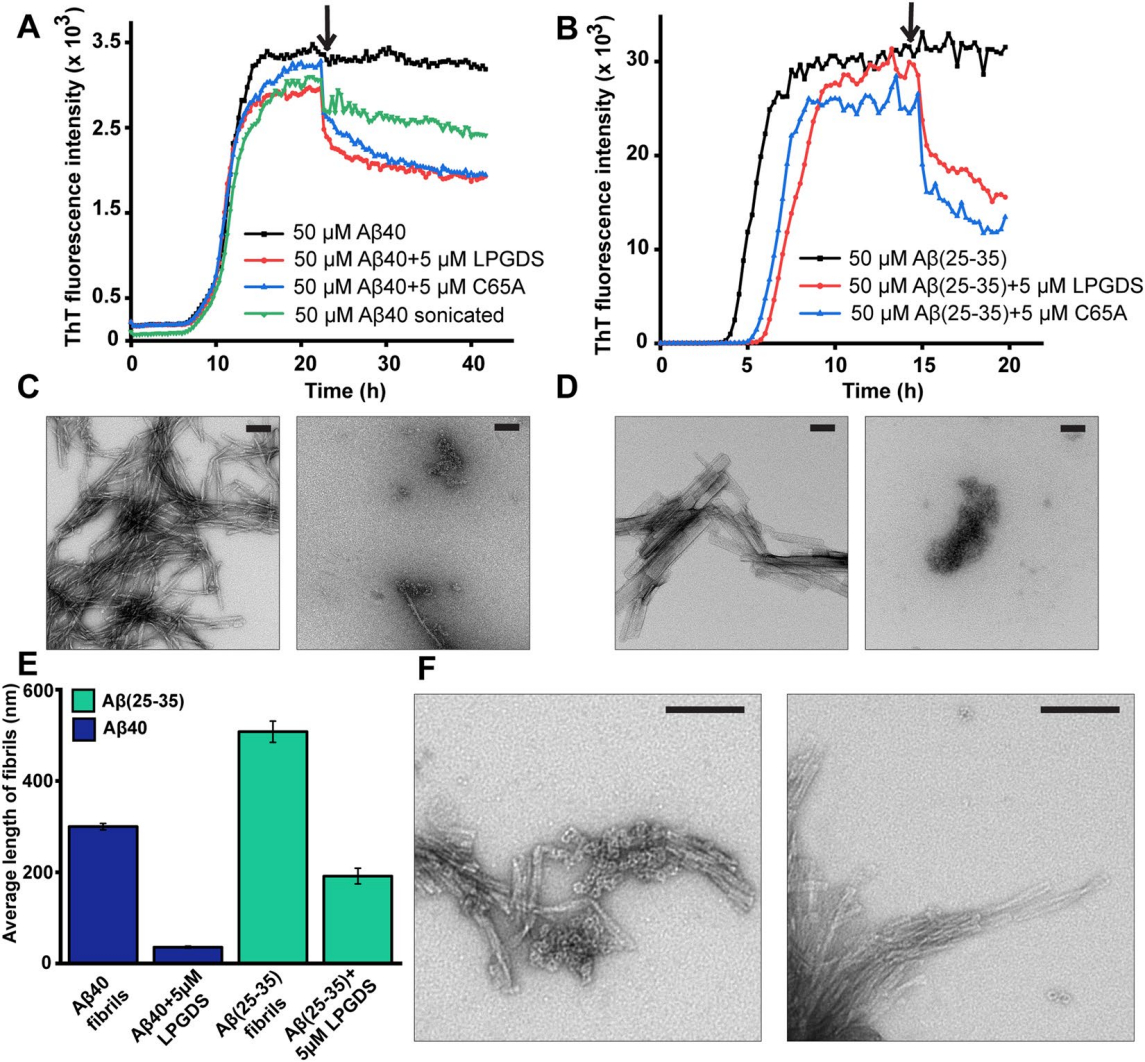
Disaggregase activity of L-PGDS on Aβ40 and Aβ(25–35) fibrils. (**A**) Thioflavin T fluorescence plotted against time for 50 µM Aβ40 control (black) treated with 5 µM WT L-PGDS (red) or 5 µM C65A mutant of L-PGDS (blue), or upon sonication (green) after 22 h (black arrow) of aggregation. (**B**) ThT fluorescence plot for Aβ(25–35) fibrils obtained from 50 µM monomers over 20 h. The curves represent Aβ(25–35) fibrils (black), fibrils with addition of 5 µM WT-L-PGDS (red) or 5 µM C65A mutant of L-PGDS (blue) at 15^th^ hour (black arrow) of incubation. The shift in the log phase onset of the aggregation might be caused by the presence of smaller aggregates which remained even after the solubilization in HFIP. (**C**) Transmission electron micrographs of mature Aβ40 fibrils alone (left) and upon treatment with WT-L-PGDS (right). (**D**) TEM micrographs showing Aβ(25–35) fibrils alone (left) and Aβ(25–35) fibrils treated with WT-L-PGDS (right). (**E**) Analysis of average length of the Aβ40 fibrils (blue) and Aβ(25–35) (cyan) fibrils from TEM images in the presence or absence of L-PGDS (n~175). S.E.M. were plotted. (**F**) TEM micrographs (Scale bar: 100 nm) to identify the localization of L-PGDS to Aβ40 fibrils. L-PGDS conjugated with ferritin nanocages (left) can be seen localized along the tips of fibrils whereas ferritin nanocages alone (right) do not interact with the fibrils.

The potential role of L-PGDS in the disaggregation of Aβ40 and Aβ(25–35) fibrils was investigated by Thioflavin T assay and TEM. ThT fluorescence of amyloid peptides incubated at 37 °C for spontaneous aggregation reached a plateau at the end of 22 h and 15 h for Aβ40 and Aβ (25–35), respectively (Fig. 1A,B). Addition of 5 µM WT-L-PGDS and mutant C65A at this time point caused a decrease in fluorescence intensity, which was further reduced over time compared to the untreated peptide controls. These observations suggest changes in the fibril morphology associated with a rapid disaggregation process. A similar effect was observed by a pulse of sonication of the preformed Aβ40 fibrils resulting in the mechanical fracturing of the filaments (Fig. 1A). Thus our results demonstrate that both WT-L-PGDS and C65A mutant can alter the morphology of Aβ40 and Aβ(25–35) fibrils by fragmenting or dismantling the fibrils.

TEM micrographs of untreated Aβ40 and Aβ (25–35) samples display long fibrils, characteristic of these peptides (Fig. 1C,D [left]). When Aβ40 and Aβ(25–35) fibrils were treated with 5 µM WT- L-PGDS, smaller amorphous structures were observed (Fig. 1C,D [right]), indicating that L-PGDS disassembles both Aβ40 and Aβ(25–35) fibrils rapidly and efficiently. TEM images were further taken for analysis in Image j software, and the average length of Aβ40 and Aβ(25–35) fibrils with and without L-PGDS treatment were measured and plotted. Fig. 1E shows that the average length of (>150 [no. of fibrils]) untreated Aβ40 and Aβ(25–35) fibrils is ~300 nm and ~500 nm whereas the average length has reduced to ~35 nm and ~190 nm for L-PGDS treated Aβ40 and Aβ(25–35) fibrils, respectively. Normal distribution of fibril length for untreated and treated samples of Aβ40 and Aβ(25–35) is depicted in Supplementary Fig. S1B,C. More representative TEM images are shown in Supplementary Fig. S2.

To identify the localization of L-PGDS on Aβ40 fibrils, L-PGDS was first conjugated with magnetoferritin nanocages, and the size of conjugates was checked using Dynamic light scattering (Supplementary Fig. S3A)^34^. As L-PGDS is relatively smaller in size than Aβ40 fibrils, the conjugation of L-PGDS with ferritin is necessary for visualization. These conjugates are enzymatically active and exhibit chaperoning effect (Supplementary Fig. S3B,C). Electron micrographs of Aβ40 fibrils incubated with L-PGDS-ferritin conjugates showed that L-PGDS preferentially congregate at the growing tips of Aβ fibrils (Fig. 1F [left]), preventing the fibril growth and possibly contributing to breaking of the fibrils. The control samples incubated with ferritin alone do not show any binding to the fibrils or cause any alteration to fibril morphology (Fig. 1F [right]).

Furthermore, Aβ40 fibrils with and without L-PGDS treatment was injected into High-performance Liquid Chromatography (HPLC) for quantification of soluble Aβ40. Samples from each peak were collected, and the molecular weight was estimated by MALDI-TOF MS. The chromatogram for untreated control and L-PGDS treated sample showed a monomer peak with A_274_ = 170 mAU and A_274_ = 275 mAU, respectively (Supplementary Fig. 4A,B). Additionally, the area under the curve calculated for the monomer peak of untreated and treated samples are 3880 and 4068, respectively. These results clearly show that the number of monomers in the L-PGDS treated sample has increased compared to the control because of the disaggregation property of L-PGDS. Additionally, the size of the aggregates in untreated and L-PGDS treated Aβ40 fibrils were determined using Dynamic light scattering (DLS) technique. The size distribution for untreated Aβ40 fibrils showed peaks at ~2000, 750 and 340 nm, whereas L-PGDS treated fibril sample showed peaks at ~970 and 270 nm (Supplementary Fig. S4C). The peak at ~4 nm in L-PGDS treated sample corresponds to the size of L-PGDS. This demonstrates that the fibrils are broken down by L-PGDS.

To further confirm the disaggregase role of L-PGDS, insoluble protein aggregates were extracted from human AD brain. Addition of L-PGDS to the insoluble aggregated protein mass resulted in solubilization of several proteins. Hexafluoroisopropanol (HFIP) and formic acid-treated samples served as positive controls. The follow-up proteomics analysis identified, at least 187 released proteins from L-PGDS-treated sample, 191 and 365 proteins from HFIP and formic acid-treated samples, respectively, (Supplementary Table. S1). L-PGDS was effective in solubilizing some critical proteins like synaptotagmin, Malate dehydrogenase, Acetyl-CoA acetyltransferase, Pyruvate kinase, L-Lactate dehydrogenase, 14-3-3 Protein ζ/δ, 14-3-3 Protein β/α, Dynein, Profilin-2 and phosphofructokinase which even formic acid and HFIP could not solubilize. L-PGDS treatment has extracted proteins such as hemoglobin, whose levels are significantly increased in AD brain where it binds to Aβ fibrils and co-localizes with amyloid plaques^35^. Proteomic analysis of L-PGDS solubilized aggregates identified proteins like α-enolase, glyceraldehyde 3-phosphate dehydrogenase, and L-lactate dehydrogenase B chain, which were previously found to be significantly elevated in AD brain^36^. Some energy metabolic enzymes such as transketolase, Acetyl-CoA acetyltransferase, malate dehydrogenase, serum albumin, and phosphofructokinase are also upregulated in the AD brain^36,37^. L-PGDS solubilized all these glycolytic and energy metabolic enzymes from the protein aggregates of AD. In addition, chaperones like HSP 90 protein, α-crystallin B chain that are commonly abundant in AD in the region of senile plaques^38^; signal transduction proteins such as 14-3-3 β/α, 14-3-3 ζ/δ, synaptotagmin, Dihydropyrimidinase-related protein 2 and profilin-2; and membrane trafficking proteins including dynamin, dynein, and clathrin were also identified in L-PGDS-treated extractions of AD brain tissues (Table 1).

**Table 1:** Comparison of proteins extracted by L-PGDS, Formic acid and HFIP from insoluble protein aggregates of AD brain to proteins commonly found in AD brain published in literature.

Protein name	Musunuri *et al*., 2014	Schonberger *et al*., 2001	Lujian Liao *et al*., 2004	Our study (Proteins extracted by L-PGDS)	Our study (Proteins extracted by HFIP)	Our study (Proteins extracted by Formic acid)
α- crystallin B chain	×	✓	×	✓	✓	✓
α- enolase	✓	✓	×	✓	×	✓
Glyceraldehyde 3-phosphate dehydrogenase	✓	✓	×	✓	×	✓
Hemoglobin	×	✓	×	✓	✓	✓
Succinyl CoA: 3-ketoacid-coenzyme A transferase	×	✓	×	✓	×	✓
Synaptotagmin	×	✓	×	✓	×	×
Ferritin	✓	×	×	✓	✓	✓
Malate dehydrogenase, cytoplasmic	✓	×	×	✓	×	×
Serum albumin	✓	✓	×	✓	✓	✓
Transketolase	✓	×	×	✓	×	✓
Acetyl-CoA acetyltransferase, mitochondrial	✓	×	×	✓	×	×
Pyruvate kinase	✓	×	×	✓	×	×
l-Lactate dehydrogenase B chain	✓	×	×	✓	×	×
14-3-3 Protein ζ/δ	✓	×	×	✓	×	×
14-3-3 Protein β/α	✓	×	✓	×	×	×
Cathepsin D	✓	×	✓	×	✓	✓
Heat shock protein HSP 90-α	✓	×	×	✓	×	✓
Heat shock 70 kDa protein	✓	×	×	×	✓	✓
Glial fibrillary acidic protein	✓	×	✓	×	✓	✓
Dynamin 1	×	×	✓	✓	×	✓
Dynein, heavy chain 1	×	×	✓	✓	×	×
Profilin-2	✓	✓	×	×	×	×
Phosphofructokinase	×	×	✓	✓	×	×
Clathrin heavy chain	✓	×	✓	✓	×	✓
Dihydropyrimidinase-related protein 2	✓	✓	×	✓	×	✓
Cofilin-1	✓	×	×	✓	✓	✓

### L-PGDS inhibits primary and secondary nucleation

**Figure 2:**
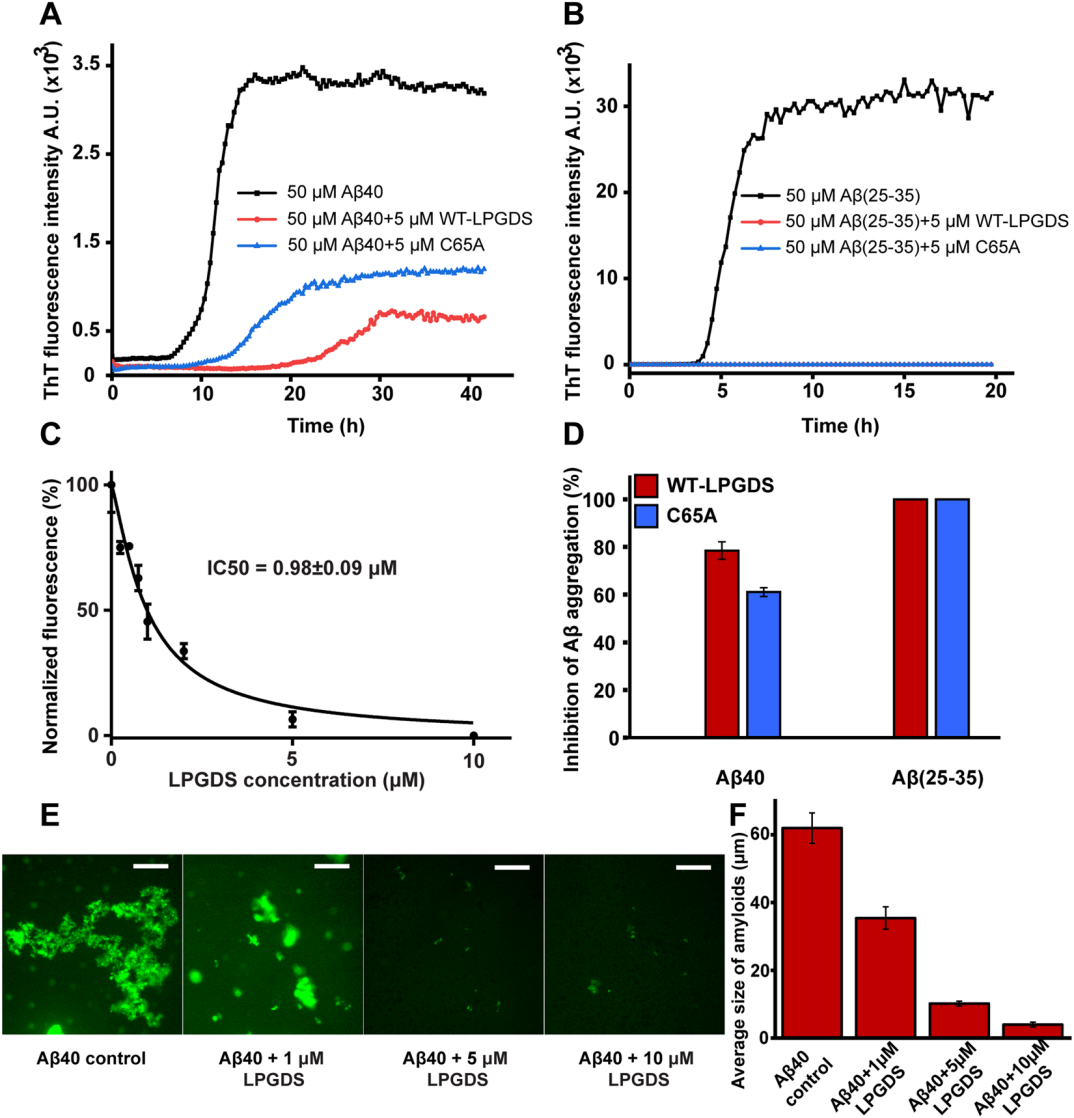
Protective role of L-PGDS as a chaperone. (**A**) Thioflavin T fluorescence plot for 50 µM Aβ40 (black) treated with 5 µM WT L-PGDS (red), 5 µM C65A mutant (blue). (**B**) Thioflavin T curve for 50 µM Aβ(25–35) (black), treated with 5 µM WT-L-PGDS (red) and 5 µM C65A mutant (blue). (**C**) IC_50_ fitting curve for L-PGDS on Aβ40 aggregation (IC_50_ = 0.98 ± 0.09 µM) (S.E.M calculated for n = 3). (**D**) Inhibition of Aβ40 and Aβ(25–35) in the presence of WT-L-PGDS (red) and C65A (blue). (**E**) Fluorescence microscopy images (Scale bar: 50 μm) of untreated Aβ40 (control) and Aβ40 incubated with 1 µM, 5 µM and 10 µM WT- L-PGDS (left to right). (**F**) Quantification of average size of the amyloids from untreated Aβ40 and 1 µM, 5 µM and 10 µM WT- L-PGDS treated samples (S.E.M calculated for n~120).

To investigate the inhibitory role of L-PGDS in Aβ aggregation, Aβ40 and Aβ(25–35) peptides were incubated at 37 °C with and without L-PGDS in ThT assay. Both Aβ40 and Aβ(25–35) displayed a characteristic sigmoidal curve indicative of amyloid formation involving primary nucleation, fibril elongation, and secondary nucleation^39^. The ThT curve showed increased fluorescence after 8 h and 4 h and reached a plateau after 14 h and 7 h for Aβ40 and Aβ(25–35), respectively (Fig. 2A,B). The increased fluorescence intensity of ThT for Aβ(25–35) is suggestive of more fibrils with extended hydrophobic surfaces for better ThT fluorescence^40^. Aβ40 peptide incubated with 5 µM WT- L-PGDS and C65A mutant exhibited significantly reduced fluorescence compared to the untreated control. To calculate the IC50 of WT- L-PGDS for Aβ40 aggregation, Aβ40 was incubated with different concentrations of L-PGDS, and the end-point fluorescence intensity was taken for the calculation. The data were normalized and fitted using (Inhibitor vs. normalized response - Variable slope) non-linear curve fitting in Graphpad prism7. ThT data used for IC50 calculation is shown in Supplementary Fig. S5C. The IC50 of WT- L-PGDS for Aβ40 aggregation was calculated to be 0.98 ± 0.09 µM (Fig. 2C). Both WT- L-PGDS and C65A treated Aβ40 samples showed ~80% and ~60% inhibition of Aβ40 aggregation respectively compared to the control, indicating that WT-L-PGDS is more effective in inhibiting aggregation than C65A mutant (Fig. 2D). Kanekiyo *et al*. stated that the C65A mutant of L-PGDS does not inhibit Aβ40 aggregation, proposing C65 to be an essential residue in the chaperone function of L-PGDS^10^. However, our results indicated that there is residual chaperone activity in the C65A mutant. Similarly, when the Aβ (25–35) peptide was incubated with 5 µM WT- L-PGDS and mutant C65A, respectively, complete inhibition of aggregation was observed, suggesting that both WT- L-PGDS and mutant C65A are capable of completely inhibiting primary and secondary nucleation. Aβ40 peptide incubated with 1 µM WT- L-PGDS and C65A mutant also showed ~40% inhibition of aggregation and even at such low concentration (Protein: Peptide (1:50)), both WT-L-PGDS and C65A exhibited complete inhibition of aggregation for Aβ (25–35) (Supplementary Fig. S5A,B).

To study the effect of L-PGDS on the microscopic events of aggregation, the ThT curves obtained for Aβ40 in the absence and presence of L-PGDS were taken for analysis using Amylofit^41^ and the curve fitting was performed using the multistep secondary nucleation dominated model with a mean residual error of 0.00158. The rate constants for primary nucleation, secondary nucleation, and elongation calculated are 4.36 × 10^−4^ M^−1^ h^−1^, 376.9 M^−1^ h^−1^, and 3.77 × 10^3^ M^−1^ h^−1^ respectively for Aβ40 control and 3.03 × 10^−5^ M^−1^ h^−1^, 59.85 M^−1^ h^−1^ and 3.6 × 10^3^ M^−1^ h^−1^ for L-PGDS treated Aβ40. Fourteen-fold reduction in primary nucleation and six-fold reduction in secondary nucleation rate constant suggests that L-PGDS inhibits the Aβ40 aggregation by targeting the monomers, oligomers and fibril surface eventually resulting in reduced fibril content compared to the untreated control.

Inhibition of Aβ40 aggregation by L-PGDS was studied morphologically by fluorescence microscopy (Fig. 2E). Aβ40 samples incubated with 1, 5, and 10 µM WT- L-PGDS were taken for analysis. Untreated Aβ40 peptide used as control exhibited long fibrils (Fig. 2E [left]) whereas Aβ40 grown with 1 μM L-PGDS showed larger amorphous structures. Aβ40 incubated with 5 and 10 μM L-PGDS displayed small amorphous structures instead of fibrils (Fig. 2E [right]). The fluorescence microscopy images were further analyzed using Image j software to measure the size of amyloids in Aβ40 control, and L-PGDS treated samples. More than 100 amyloid structures were taken for analysis in each sample. The analysis showed an average size of ~60 µm for untreated Aβ40 control and ~40, 10, and 5 µm for 1, 5, and 10 µM L-PGDS treated samples, respectively (Fig. 2F). This confirms the concentration-dependent inhibition of Aβ40 fibrillation by L-PGDS. The normal distribution of amyloid size in each sample plotted using Origin pro-2018 is shown in Supplementary Fig. S1A. More representative fluorescence images are shown in Supplementary Fig. S5D. Our observations suggest that L-PGDS affects both the aggregation kinetics and the morphology of Aβ aggregates.

### L-PGDS specifically binds Aβ40 in 1:1 stoichiometric complex in a manner distinct from catalytic substrates

Using NMR and X-ray analysis, our previous studies have demonstrated that L-PGDS binds to two substrate-analog molecules at the primary (catalytic) and secondary (peripheral) binding sites, respectively, with different affinities^31^. MALDI-TOF mass spectrometry (MS) analysis shows that L-PGDS-Aβ40 complex (in 1:1 stoichiometry) is sufficiently stable to withstand desorption and ionizing conditions created during this detection process (Supplementary Fig. S6). Based on the MS results, we posit that Aβ40 binds to L-PGDS in 1:1 stoichiometric ratio. In addition to L-PGDS peak at around 19.8 KDa, an additional peak at 24.1 KDa for L-PGDS-Aβ40 complex strongly supports that one Aβ40 peptide binds per molecule of L-PGDS. Although higher peaks were observed due to the propensity of L-PGDS to form oligomers through disulfide linkages, no additional peaks corresponding to L-PGDS-Aβ40 complex were detected. This observation, together with the analysis of SAXS diffraction data indicate a thermodynamically stable complex being formed at this stoichiometry.

**Figure 3:**
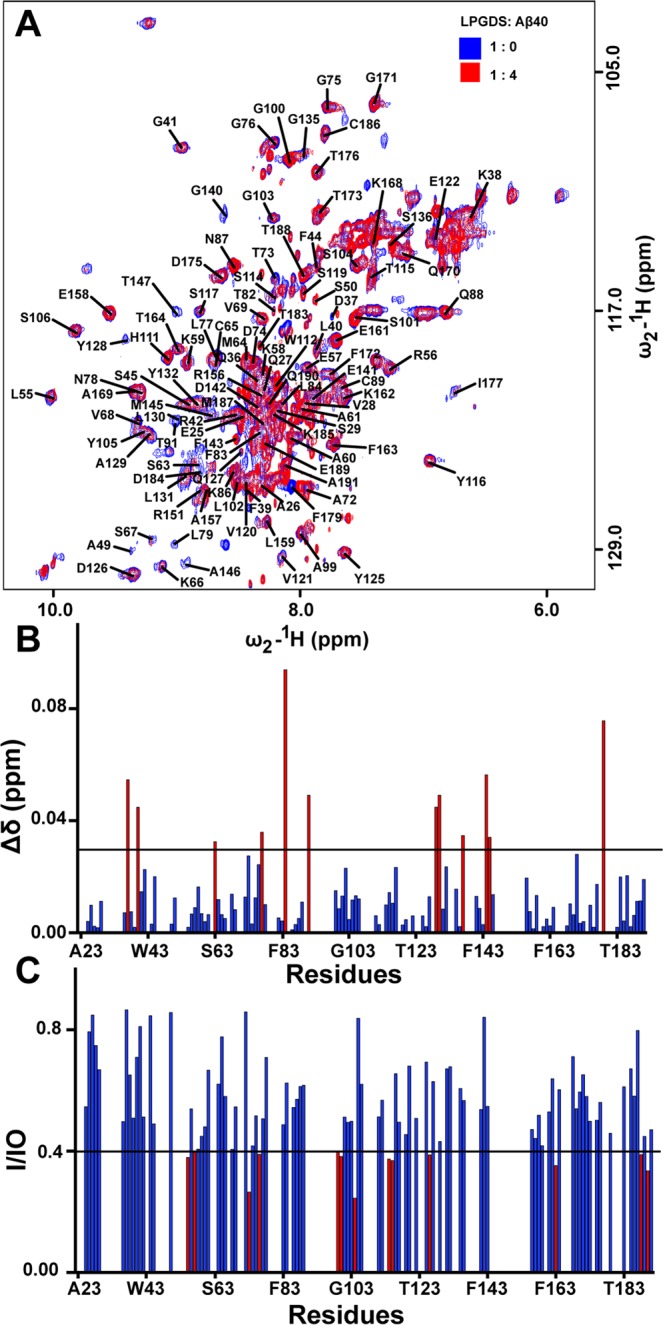
NMR titration of ^15^N-labeled L-PGDS with unlabeled Aβ40. (**A**) Superposition of ^1^H-^15^N HSQC spectra of L-PGDS alone (blue) and upon addition of Aβ40 (red) at a molar ratio of 1:4. The cross-peaks were assigned following^31^. (**B**) Chemical shift perturbation plot of L-PGDS showing residues with CSP > 0.03 highlighted in red. (**C**) Bar graph showing changes in intensity ratio (I/Io) comparing L-PGDS alone and L-PGDS-Aβ40 complex. Residues showing I/Io < 0.4 are highlighted in red. Io is the intensity of free protein and I is the intensity of the complex.

NMR in solution was used to delineate key residues involved in the binding interface of WT- L-PGDS-Aβ40 complex. A 2D ^1^H-^15^N HSQC spectrum of WT- L-PGDS was recorded in the absence and presence of Aβ40 peptide at a molar ratio of 1:4 (protein: peptide) (Fig. 3A). Upon addition of the Aβ40 peptide to ^15^N-labeled L-PGDS, cross-peaks stemming from residues D37, L40, S63, L77, L84, T91, A129, L130, K137, R144, M145 and F179 were shifted (Fig. 3B), while cross-peaks from residues L55, E57, T73, G76, A99, G100, S104, S114, T115, D126, F163, T188, and Q190 showed significant attenuation in signal intensity (Fig. 3C). Also, cross-peaks from residues A49, N51, S67, L79, T82, T91, S114, V121, T123, Y128, L130, K137, G140, M145, A146, T147, K160, I177, and D184 completely disappeared upon binding. The catalytic and proximal residues are not affected by binding. In contrast, titration of ^15^N-labeled WT-L-PGDS with the substrate analogue resulted in chemical shift perturbation of residues D37, F39, W43, A49, W54, R56, E57, S67, M64, C65,W112, Y116, Y128, G140, D142, R144, T147, L148 and significant enhancement of cross-peak intensities in residues T73, G75, G76 and L77, indicating different binding modes between two ligands^31^. The exact cause of the excessive line broadening observed in L-PGDS-Aβ40 titration is not well-established but can be tentatively attributed to the fast complex dissociation rate resulting in an intermediate exchange regime in the NMR chemical shift time scale^42^. The small and widespread chemical shift perturbations observed in the spectrum could be due to the formation of an encounter complex^43^ comprising an ensemble of Aβ40 orientation within the L-PGDS-Aβ40 complex.

**Figure 4:**
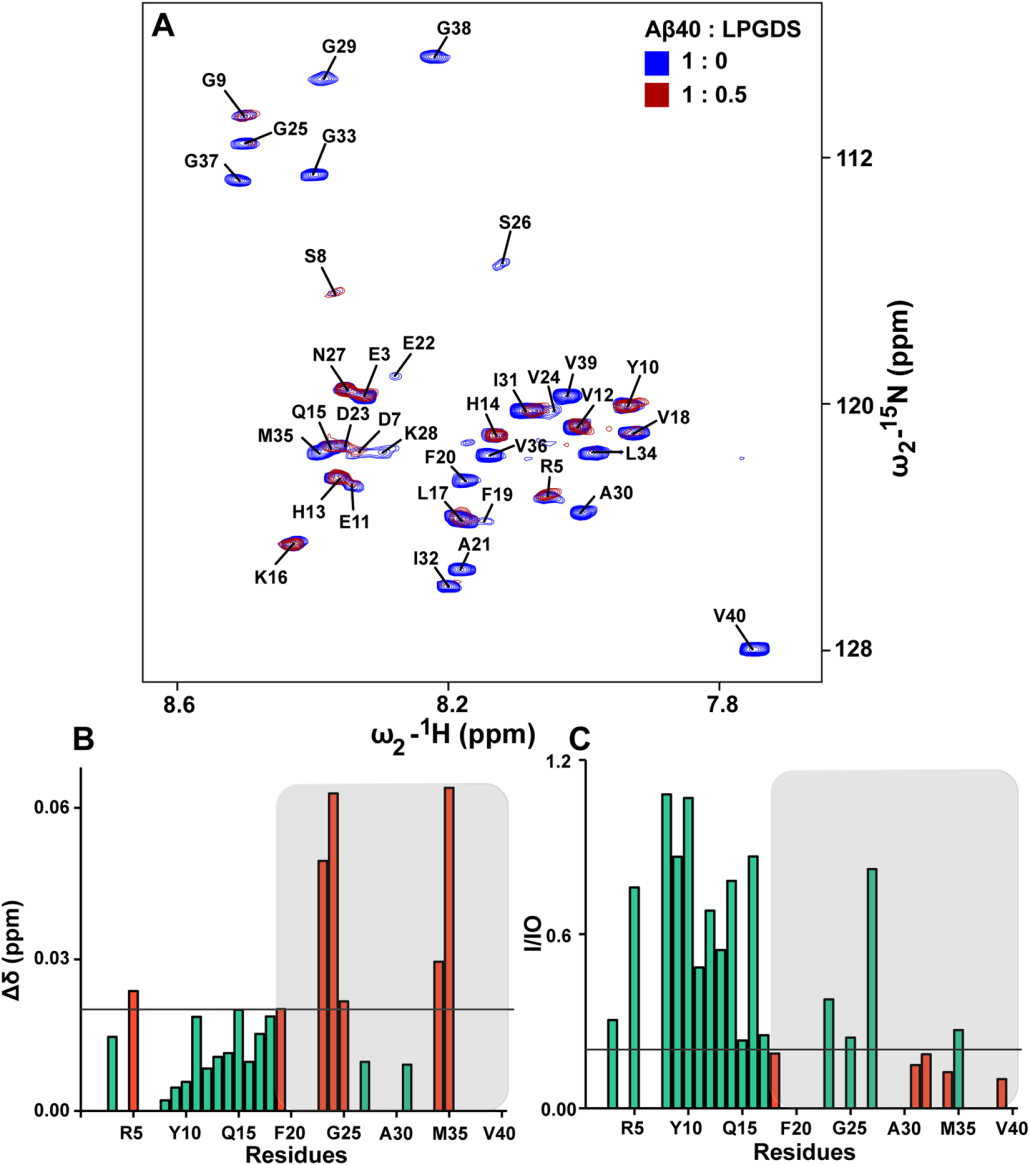
NMR titration of ^15^N-labeled Aβ40 with unlabeled L-PGDS. (**A**) Superposition of ^1^H-^15^N HSQC spectra of free Aβ40 (blue) titrated with unlabeled L-PGDS (red) at a molar ratio of 1:0.5. Resonance assignments of monomeric Aβ40 spectrum was obtained from Biological magnetic resonance data bank (BMRB) entry 11435. (**B**) Chemical shift changes between the free Aβ40 peptide and L-PGDS-Aβ40 complex. Residues with CSP > 0.02 are colored in orange (**C**) Bar plot representing the attenuation of cross-peak intensity ratio (I/Io) of free peptide (Io) and the peptide-protein complex (I). Residues with I/Io < 0.2 are colored in orange.

To investigate the binding of L-PGDS to Aβ40, we performed reverse NMR titration in which ^1^H-^15^N HSQC of ^15^N-labeled monomeric Aβ40 peptide was recorded with and without the addition of unlabeled L-PGDS at a molar ratio of 1:0.5 (Fig. 4A). Resonance assignments of monomeric Aβ40 spectrum were transferred from biological magnetic resonance data bank (BMRB) entry 11435^44^. Cross peaks from residues R5, D23, V24, L34 and M35 showed chemical shift perturbation (>0.02 ppm) (Fig. 4B) while residues V18, F19, F20, A21, E22, D23, V24, G25, S26, K28, G29, A30, I31, I32, G33, L34, M35, V36, G37, G38, V39, and V40 showed significant reduction in signal intensity (Fig. 4C). The chemical shift perturbations and line broadening of residues in both L-PGDS and Aβ40 peptide are suggestive of a complex formation. From our NMR titrations, it is evident that the hydrophobic C-terminus of Aβ40 (residues 18–40) is primarily involved in binding to L-PGDS which is well in agreement with the SPR results shown previously^10^. Due to extensive line broadening, direct structure determination of the complex by NMR was not possible.

### Binding of Aβ40 to L-PGDS adds an extra domain

**Figure 5:**
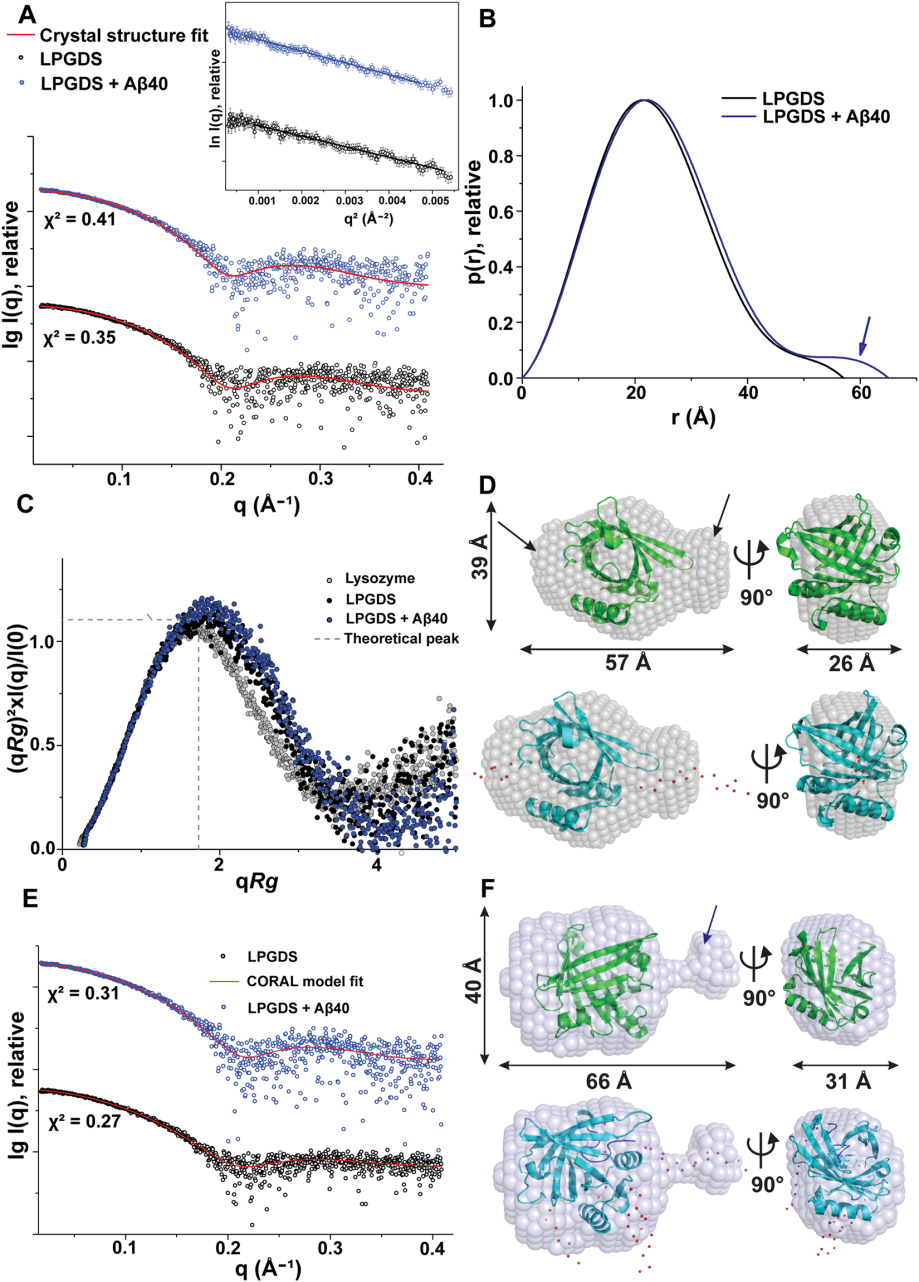
Solution X-ray scattering studies of L-PGDS with Aβ40 peptide. (**A**) Experimental scattering pattern (⚬) and calculated scattering profile of the crystal structure (—; red) of L-PGDS (black) and in complex with Aβ40 peptide (blue). (Inset) Guinier plots show linearity, indicating no aggregation. The scattering profiles are offset for clarity by applying arbitrary scale factors. (**B**) Overlapping of pair-distance distribution function *P(r)* of L-PGDS (black), in complex with Aβ40 peptide (blue). L-PGDS with Aβ40 has an extended tail (represented by the blue arrow). (**C**) Normalized Kratky plot of L-PGDS (•; black) compared to its complex and the compact globular lysozyme (•; grey) with a peak (—; grey), representing the theoretical peak and assuming an ideal Guinier region of a globular particle. (**D**) The averaged and filtered envelope of L-PGDS (grey) from ten independent *ab initio* reconstructions using DAMMIN superimposed (top) onto the cartoon representation of the crystal structure (green; PDB ID: 4IMN) and (bottom) with the CORAL model (cyan). The flexible N- and C-terminal residues in L-PGDS are shown in red. The unoccupied density is represented by an arrow. Front (left) and side (right) views are displayed. (**E**) Fitting of the CORAL model (—; red) to the experimental scattering pattern (○) for L-PGDS (black) and the L-PGDS-Aβ40 complex (blue). (**F**) The averaged and filtered *ab initio* low-resolution shape of L-PGDS with Aβ40 (blue) superimposed (top) onto the cartoon representation of the crystal structure (green; PDB ID: 4IMN) and (bottom) with the CORAL model (cyan). The flexible N- and C-terminal residues in L-PGDS are shown in red and for Aβ40 in magenta.

To track the overall shape change of L-PGDS in 1:1 complex with Aβ40, SAXS in solution was performed, which provides information about the radius of gyration (*Rg*), maximum particle dimension (*Dmax*), low-resolution shape, conformation, and assembly state. L-PGDS measured at 1.0, 2.5 and 4.0 mg/ml showed neither a concentration-dependent increase in particle size nor aggregation as inferred from the Guinier plot (In I(q) vs. q^2^), which appeared linear and revealed good data quality (Supplementary Fig. S7A). From the slope of the linear fit, the experimental *Rg*-values derived at a protein concentration of 2.5 mg/ml is 17.91 ± 0.32 Å (Supplementary Table. S2; Fig. 5A). The extended scattering curve is converted using the indirect Fourier transform to provide the distance distribution function (*P[r]*), which is a histogram of distances between all possible pairs of atoms within a particle. The *P(r)* of L-PGDS exhibits a single local maximum with a slightly right-skewed distribution (Fig. 5B) with a *Dmax* of 57 ± 5 Å, indicating a globular protein with slight elongation in solution. The *Rg*-value extracted from the *P(r)* function (18.04 ± 0.17 Å) agreed with the *Rg*-value derived from the Guinier region (Supplementary Table. S2). The scattering curve is transformed into a normalized Kratky plot^45^. L-PGDS exhibits the profile typical of standard globular proteins (Fig. 5C). The Porod-Debye plot was generated by processing scattering data as *q*^4^*∙I(q)* vs. *q*^4^, which displayed a plateau, typical for a compact molecule (Porod exponent being 3.9) (Supplementary Fig. S7D). The molecular mass calculated for L-PGDS based on the volumes extracted from a higher angle of scattering data, the excluded volumes and the volume of correlation, reveal that human L-PGDS exists as a monomer in solution at the tested range of concentrations (Supplementary Table S2). The theoretical scattering curve for the crystallographic structure (PDB ID: 4IMN) computed using CRYSOL^46^ had a good fit to the experimental scattering data with a discrepancy (χ^2^) of 0.35 (Fig. 5A). Ten low-resolution shapes of human L-PGDS were reconstructed *ab initio*, which had a normalized spatial discrepancy (NSD) of 0.52 ± 0.02. The final averaged and filtered model was superimposed on the crystal structure with an NSD of 1.05 Å (Fig. 5D). The shape of L-PGDS has a larger globular domain and a short domain. The unoccupied density in the bead model (represented by the arrow in Fig. 5D) suggested that the N and C-terminal residues, which are not reconstructed in the crystal structure, might be flexible in solution and occupy the extra density. To model the missing seven N-terminal and fifteen C-terminal residues, rigid body modelling was performed. The CORAL model had a better fitting to the experimental data (χ^2^ = 0.27) than the crystal structure (χ^2^ = 0.35) and was superimposed on the SAXS shape with an NSD of 1.09 (Fig. 5D,E).

Addition of Aβ40 to L-PGDS resulted in a stable complex, and no aggregation of Aβ40 was observed as demonstrated by the Guinier plot (Fig. 5A; inset). Nevertheless, the overall particle size of the complex increased by approximately 1 Å, resulting in an *Rg*-value of 18.91 ± 0.29 Å (Supplementary Table. S2). Interestingly, the overall length increased by 8 Å, leading to a *Dmax* of 65 ± 5 Å (Supplementary Table. S2). This difference is reflected in the *P(r)* profile, where a long tail with a small hump is observed, indicating the presence of a small additional domain (Fig. 5B). Although the normalized Kratky plot of L-PGDS with Aβ40 is similar to the protein alone, a slight shift is observed, suggesting a more elongated shape (Fig. 5C and Supplementary Fig. S7C). Moreover, the Porod-Debye plot showed a plateau with Porod exponent of 3.9, similar to protein alone, indicating a compact molecule (Supplementary Fig. S7D). An *Ab initio* low-resolution shape of L-PGDS with Aβ40 (NSD = 0.54 ± 0.02) showed two domains; one large globular domain and an elongated protrusion (Fig. 5F) with the extra density assigned to the Aβ40 peptide. Using the crystallographic structure of Anticalin, a close homologue of L-PGDS, in complex with Aβ40 (visible residues 16–28, PDB ID: 4MVI)^47^ as a template, a CORAL model was generated allowing flexibility for the N- and C-terminal residues of the Aβ40 peptide (15 residues in N-terminus and 12-residues in C-terminus) and for the L-PGDS (7 and 15 residues in N- and C-termini, respectively). This CORAL model shows an improved fit to the experimental data (χ^2^ = 0.31) and can be superimposed to the SAXS shape with the NSD of 0.9 (Fig. 5E,F). In this model, the N-terminal residues of Aβ40 occupy the extra elongated domain (Fig. 5F) with the C-terminal residues of the peptide and the N- and C-terminals of L-PGDS positioned inside the larger globular part. The normal *P(r)* distribution, the peak at the theoretical value in the normalized Kratky plot, a plateau in the Porod-Debye plot, a smaller NSD value between the *ab initio* shapes and a good fit to a CORAL model indicates, that the L-PGDS-Aβ40 complex is compact and rigid, hence a single structure was derived for the complex.

### MD simulation model of L-PGDS/Aβ40 complex

**Figure 6:**
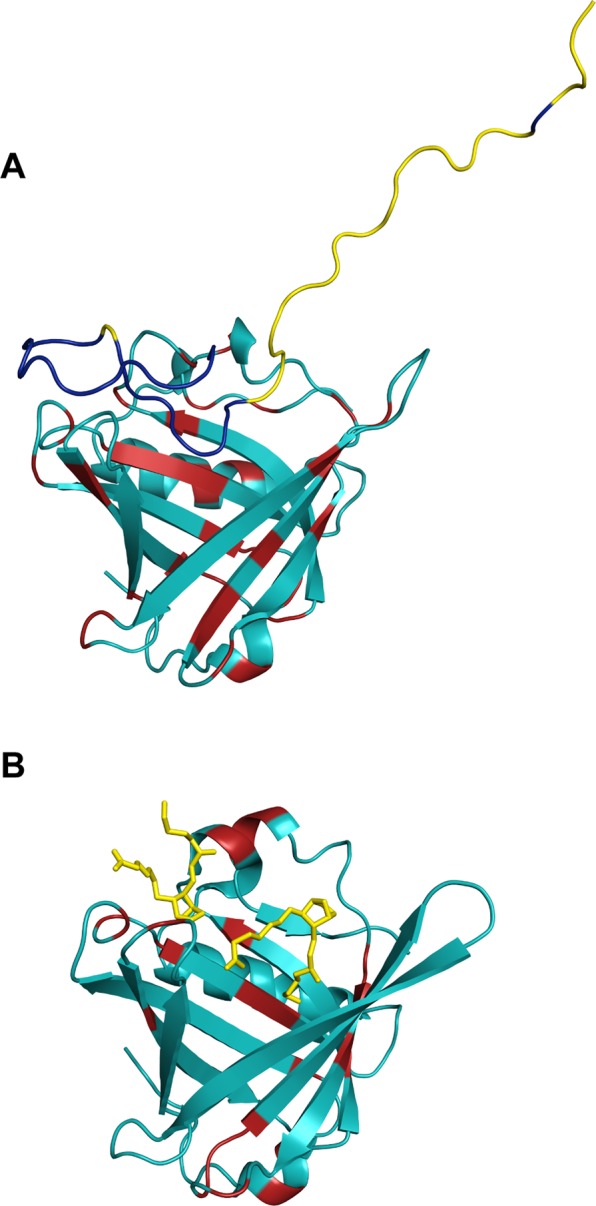
Model of L-PGDS in complex with Aβ40 and L-PGDS-substrate analog complex. Model of L-PGDS bound with Aβ40 obtained from molecular dynamics simulation. Structure of L-PGDS is represented in cyan and Aβ40 is represented in yellow. Residues which showed chemical shift perturbation or line broadening upon binding as shown by NMR are highlighted in red and blue in L-PGDS and Aβ40, respectively (**B**) X-ray structure of the L-PGDS-substrate analog complex from Lim *et al*. L-PGDS structure is shown in cyan and the substrate analog is represented in yellow. Red highlights are the residues which showed chemical shift perturbation or signal broadening upon binding with the substrate analog^31^.

The representative model of the L-PGDS/Aβ40 complex was reconstructed using 5 repeats of classical molecular dynamics simulations with random different initial velocities to increase sampling of the conformation space. The radius of gyration plot for Aβ40 throughout the five repeats shows a diverse range and dynamic fluctuation between 1.0 and 2.4 nm (Supplementary Fig. S8A) which reflects the conformational flexibility of Aβ40 as an intrinsically disordered peptide. We extracted structures corresponding to conformations of Aβ40 with a radius of gyration values larger than 1.95 nm sampled in the simulations. Geometric clustering performed for Aβ40 with a clustering cutoff of 0.8 nm resulted in seven clusters, with the top five clusters accounting for more than ~99% of the conformations. The representative structure of cluster 2, which accounts for ~12% of the conformations, shows good agreement with experimental data (Fig. 6A). Besides, the N-terminal residues 1–16 of Aβ40 did not interact with the chaperone and are extended as seen in the space-filling model of SAXS (Fig. 5F) while the residues 18–26 are located at the entrance of L-PGDS calyx. The residues 29–40 were transiently packed against a shallow grove on the L-PGDS surface, exhibiting considerable conformational variation in packing. A comparison between L-PGDS-Aβ40 complex model and L-PGDS-substrate crystal structure shows that Aβ40 binding to L-PGDS is different from substrate binding (Fig. 6B).

To further understand the interaction between L-PGDS and Aβ40, we generated a contact map based on the average heavy atom distances between residues of L-PGDS and Aβ40 from the simulation representative cluster, with a range of 0 (white) to beyond 10 Å (dark blue) (Supplementary Fig. S8B). On the contact map, we were able to identify several light-colored regions, which is indicative of L-PGDS and Aβ40 residues being in proximity to each other (Supplementary Fig. S8B). Residues in L-PGDS and Aβ40 that showed chemical shift perturbation (CSP) or line broadening in HSQC spectrum upon binding are shown in red and yellow boxes, respectively. Residues which showed perturbation or signal broadening in NMR were also found in the light-colored regions of the residue contact map. This indicates that our model agrees with experimental NMR data, showing a direct binding between L-PGDS and Aβ40 (Fig. 6A, Supplementary Fig. S8B).

Moreover, our simulation model can propose additional contact sites between L-PGDS and Aβ40 (Magenta, Supplementary Fig. S8B). It is important to note that in addition to direct binding, CSP can also arise as a result of conformational changes in the protein that were induced during ligand binding^48^. Few residues that showed CSP were found deep inside the L-PGDS calyx (Fig. 6A). We attribute the presence of such CSP and broad loss of resonances as a result of allosteric changes in L-PGDS upon binding with Aβ40.

## Discussion

Molecular chaperones play a protective role in neurological disorders by inhibiting or modulating protein aggregation^49^. Among the several proteins studied for their chaperone function, HSP60 inhibits aggregation by acting upon the early oligomeric species, which acts as seeds in the aggregation process^50^. The extracellular chaperone, clusterin sequesters Aβ oligomers and αB-crystallin prevent elongation of Aβ fibrils in the human brain^51,52^. Human Brichos domains specifically inhibit the secondary nucleation in Aβ aggregation and limit human Aβ42 toxicity^53^. While some of these proteins may function as important Aβ chaperones contributing to the maintenance of Aβ systemic homeostasis, the search for a major brain-specific chaperone prompted us to study the mechanism of chaperone activity of L-PGDS in both inhibition of Aβ fibril formation and disaggregation.

L-PGDS inhibits primary and secondary nucleation of Aβ40 by interacting with monomers and fibril surface and thereby reduces the final fibril content. Our NMR result reveals specific binding of L-PGDS to the C-terminus of Aβ40, which includes the region G25-G29, the critical residues involved in the conformational change of Aβ from random coil to β-sheet structure^54^. Interactions with these residues might be the cause of inhibition. This result agrees well with the SPR result shown previously^10^. The mass spectrometry analysis shows that one molecule of Aβ40 is bound to the L-PGDS calyx, forming a stable complex. However, Aβ40 peptide in this complex retains a significant degree of conformational flexibility, potentially adopting a range of conformations including extended beta-hairpin as observed in anticalin/Aβ40 or short helices predicted by the Hamiltonian replica-exchange MD simulations (data not shown).

Our results show that L-PGDS inhibits Aβ40 and Aβ(25–35) aggregation effectively even at a molar ratio of 1:10, which is in agreement with previous studies^10^. The concentration of L-PGDS in CSF under normal condition (26 µg/ml) would be sufficient to prevent aggregation and keep the Aβ40 (1.6–17.1 ng/ml) in a monomeric state^11,55^. However, recent studies have shown that the level of L-PGDS in the CSF of AD patients is significantly reduced as compared to healthy controls^56,57^. Though several other chaperones such as Hsp60, Brichos, and αB-crystallin have been shown to inhibit Aβ aggregation^50,58,59^, the levels of L-PGDS in CSF is much higher, elevating its impact on the Aβ homeostasis^11,60^.

Additionally, L-PGDS exhibited characteristics of a disaggregase by dismantling preformed fibrils of Aβ40 and Aβ(25–35) as shown in ThT assay, TEM images, HPLC, DLS, and proteomics data. Although the precise molecular mechanism remains elusive, here we explained the possible mechanism by which L-PGDS breaks the fibrils. L-PGDS has established specific interaction with the region G25-G29, the exposed residues in fibrils that contain the bend which connects two β-sheets^54^. We hypothesize that this interaction might potentially disrupt the non-covalent intermolecular interactions between the fibrils and disassembles them. Similar effects observed for fibril disintegration by L-PGDS and sonication suggests that direct interaction of L-PGDS with amyloid fibrils (Fig. 1F) might result in fibril remodeling rather than passively shifting the monomer/fibril equilibrium by sequestering the monomers. With adequate binding interactions, L-PGDS can disaggregate Aβ40 fibrils without co-chaperones and ATP consumption; a mechanism similar to the molecular chaperone CpSRP43 (Chloroplast signal recognition particle)^61^. HTRA1 is a yet another molecular chaperone which executes disaggregation without the aid of any co-chaperones and ATP consumption^62^. HTRA1^S328A^, a proteolytically inactive variant of HTRA1, has shown to disintegrate preformed fibrils of 4 R tau when treated at an equimolar ratio in sedimentation assay^63^. Quantification of tau fibrils disintegration by HTRA1^S328A^ showed a fourfold reduction in the amount of fibrils compared to the untreated control, and the AFM image of HTRA1^S328A^ treated fibrils still showed residual fibrils or aggregates^63^.

L-PGDS exhibits disaggregase activity for Aβ40 and Aβ(25–35) fibrils even at sub-stoichiometric ratios of 1:10. Moreover, L-PGDS efficiently solubilized several key proteins commonly found in AD brain proteome when added to the insoluble protein aggregates extracted from AD brain tissue. Proteomic analysis of L-PGDS solubilized plaque proteins resulted in the identification of 187 proteins (Supplementary Table. S1), which includes chaperone proteins and proteins of different pathways including glycolysis, energy metabolism, and signal transduction. These results further reinforce the disaggregase role of L-PGDS.

Currently, 17 key human proteins and protein complexes have been identified for their possible function as neuroprotective amyloid-β chaperones (Supplementary Table. S3). The corresponding mode of action of these chaperones (inhibit primary or secondary nucleation, disaggregate bundle of amyloids, delay fibril growth), ATP dependence, location (intracellular or secreted), levels of expression in various brain areas (cerebral cortex, hippocampus, caudate, cerebellum)^64^ vary significantly, reflecting their relative contribution to the neuroprotection. L-PGDS is a unique chaperone, ubiquitously expressed in various parts of the brain, which inhibits primary and secondary nucleation, and disaggregates preformed fibrils of Aβ without ATP consumption. In conclusion, the novel disaggregase property of L-PGDS described in this paper elucidates the mechanism for its previously suggested role as a chaperone.

## Methods

### Protein expression and purification

The gene encoding Human wild-type L-PGDS was cloned in pNIC-CH2 vector containing the C-terminal hexa-histidine tag. 1–22 amino acids encoding the signal peptide was truncated from the gene sequence. The plasmid was then transformed into Rosetta 2 DE3 singles *E*. *coli* cells (Novagen) for expression. Cells were grown at 37 °C in terrific broth and induced at OD_600_ = 0.8–1.0 with 1 mM Isopropyl β-D-1-thiogalactopyranoside (IPTG) and incubated at 37 °C for 3–4 h. For ^15^N labeled protein expression, cells were grown in M9 media containing 1 g/L ^15^N labeled ammonium chloride and induced with 0.5 mM IPTG followed by incubation at 18 °C overnight. His-tagged protein was purified using Ni-NTA resin through immobilized metal affinity chromatography (IMAC). Protein collected was entirely denatured with 8 M urea and then refolded by dialysis at 4 °C to get rid of any hydrophobic molecules attached to the hydrophobic pocket of L-PGDS. The protein was then further purified by Superdex75 10/300 GL column in AKTA Fast performance liquid chromatography (FPLC) in 20 mM HEPES, 150 mM NaCl, 2 mM TCEP (pH 6.5).

### Recombinant expression of Aβ40

pET 28a vector carrying Aβ40^65^ gene was transformed to Rosetta 2 DE3 singles *E*. *coli* cells (Novagen). Cells grown at 37 °C were induced with 0.5 mM IPTG after the OD_600_ reached 0.6–0.8, followed by incubation at 37 °C for 4 h. Inclusion bodies were extracted from the cells to get the pure peptide. The cells were lysed using sonicator for several cycles to remove soluble proteins. After several washes in buffer, the inclusion bodies were dissolved in urea and dialyzed against water. The precipitate was lyophilized and was further purified in a C18 column in *Waters*, High-performance liquid chromatography at a flow rate of 2 ml/min with a linear gradient of Acetonitrile/water containing 0.1% v/v Trifluoroacetic acid (TFA). The purity of the peptide was checked by mass spectrometry. Synthetic Aβ(25–35) and Aβ40 were purchased from China Peptides (Suzhou, China).

### Conjugation of L-PGDS to ferritin nanocages

1.6 mg/ml magnetoferritin nanocages (kindly provided by Dr. Sierin Lim, NTU) were activated for carbodiimide conjugation by adding a 1:2.5 mixture of 1-ethyl-3-(3-dimethylaminopropyl) carbodiimide hydrochloride) (EDC) and N-hydroxysulfosuccinimide (sulfo-NHS) in MES buffer and incubated at room temperature while shaking for 90 minutes in dark. 50 µM L-PGDS in 20 mM HEPES buffer with 100 mM NaCl, 2 mM TCEP (pH 7.5) was added to this mixture in equal volume and left for overnight incubation in dark with gentle shaking. Crosslinking reaction was quenched by adding Tris (pH 7.5) at a final concentration of 150 µM. These conjugates were passed through IMAC column and PD10 desalting columns to remove excess unbound ferritin nanocages.

### Preparation of monomeric peptides

Lyophilized Aβ peptides were treated with 1,1,1,3,3,3-hexafluoro-2-propanol (HFIP) and incubated at room temperature to break any preformed aggregates. The excess HFIP was then removed by lyophilization, and the dried peptide film was dissolved in a small volume of 100 mM NaOH and subsequently diluted with the buffer.

### Thioflavin T assay

Inhibition and disaggregation of Aβ aggregation by L-PGDS was studied using ThT (Sigma), a fluorophore that specifically binds to the β-sheet structure of amyloids and shows enhanced fluorescence. To study inhibition, monomeric Aβ40 and Aβ(25–35) peptides were prepared in buffer containing 20 mM HEPES, 100 mM NaCl, 2 mM TCEP (pH-7.5) at a final concentration of 50 μM Aβ40 and Aβ (25–35) and 20 μM ThT with or without 1 μM and 5 μM of WT-L-PGDS or C65A mutant. The disaggregase activity of L-PGDS was investigated by adding 1 μM and 5 μM of WT-L-PGDS or C65A mutant to the preformed fibrils of Aβ40 and Aβ (25–35). To compare the effect of L-PGDS with mechanical breakage of fibrils, Aβ40 fibrils was sonicated in a bath sonicator for 2–3 min. The Aβ control curves in Fig. 1A,B are identical to the control curves in Fig. 6A,B respectively because the same aliquots of Aβ40 and Aβ (25–35) were used as the control for both inhibition and disaggregase assay. Experiments were carried out in triplicates in NUNC 96 well black plate with incubation at 37 °C in TECAN infinite M200 Pro microplate reader with orbital shaking before each measurement. The plate was sealed completely to prevent evaporation, and measurements were read with an excitation wavelength at 440 nm and emission at 485 nm. IC50 curve fitting was performed in Graphpad prism7.

### Fluorescence and transmission electron microscopy

To study the inhibitory effect of L-PGDS, 50 μM of Aβ40 with or without 1, 5, and 10 μM L-PGDS was incubated at 37 °C for 60 hrs with 20 uM ThT in the buffer containing 20 mM HEPES, 100 mM NaCl, 2 mM TCEP (pH 7.5). Aggregation was monitored in TECAN infinite M200 Pro microplate reader. 10 µl of each sample was applied on a glass slide, and images were acquired by fluorescence microscope (Olympus microscope with Cool SNAP^HQ2^ camera) with 10x magnification.

To establish the disaggregase activity of L-PGDS, 50 μM of Aβ40 and Aβ(25–35) was incubated at 37 °C for 60 h in buffer containing 20 mM HEPES, 100 mM NaCl, 2 mM TCEP (pH 7.5). Aβ samples treated with and without L-PGDS were applied on copper-rhodium 400 mesh grids with 15 nm carbon coating (thickness) (prepared in-house) followed by negative staining with 2% uranyl acetate and then air-dried. The samples were then viewed under FEI T12, 120 kV Transmission electron microscope equipped with a 4 K CCD camera (FEI) between 48000X to 68000x magnification under low dose conditions.

### HPLC and DLS analyses of fibril disaggregation

To quantify the amount of soluble Aβ40 in the untreated and Aβ40 fibrils treated with L-PGDS, the samples were injected into Agilent Zorbax300SB-C8 column connected to Agilent tech 1260 Infinity system and ran at the flow rate of 2 ml/min with a linear gradient of Acetonitrile/water containing 0.1% v/v TFA. The area under the curve was measured by manual peak integration. Dynamic Light Scattering (DLS) analysis was used to determine the size distribution of untreated and L-PGDS treated Aβ40 fibrils. The measurements and data analysis were carried out using a Malvern Zetasizer Nano series instrument.

### NMR titration

NMR spectroscopy experiments were carried out in Bruker Avance 700 MHz with triple resonance z-axis gradient cryoprobe and Bruker DRX 600-MHz spectrometer equipped with a cryoprobe at 298 K. Uniformly ^15^N labeled L-PGDS, and Aβ40 samples were prepared in 20 mM HEPES, 150 mM NaCl, 2 mM TCEP (pH-6.5) at a concentration of 0.25 mM and 0.2 mM respectively with 5% D_2_O. To identify the key residues of L-PGDS involved in Aβ40 binding, two dimensional ^1^H-^15^N HSQC spectra of ^15^N L-PGDS and unlabeled Aβ40 were recorded at molar ratios of 1:0 and 1:4. Similarly, to detect the important residues on Aβ40, we recorded ^1^H-^15^N HSQC spectra of ^15^N Aβ40 with unlabeled L-PGDS at molar ratios of 1:0 and 1:0.5. Spectra were referenced with respect to 4, 4- dimethyl-4-silapentane-1-sulfonic acid (DSS) and then overlapped to check if the cross-peaks showed any shift or change in signal intensity. Freshly prepared monomeric Aβ40 was used immediately in both the titrations to ensure that the majority population is monomers. Data were processed in Topspin 3.5 (Bruker Corporation) and then analyzed using Computer-aided resonance assignment (CARA) (www.nmr.ch)^66^. Chemical shifts were calculated using the equation:

$${\rm{\Delta }}\omega ={\{[{\rm{\Delta }}\omega {({}^{1}{\rm{H}})}^{2}]+{[0.25\times {\rm{\Delta }}\omega ({}^{15}{\rm{N}})]}^{2}\}}^{1/2}$$

### Small-angle X-ray scattering (SAXS) analysis

The SAXS data were collected with a BRUKER NANOSTAR SAXS instrument equipped with a Metal-Jet X-ray source (Excillum, Germany) and VÅNTEC 2000-detector system^67^. The scattering patterns were measured using a sample at a detector distance of 0.67 m and a wavelength of λ = 1.3414 Å, which covered a range of momentum transfer of 0.016 < q < 0.4 Å^−1^ (q = 4π sin(θ)/λ, where 2θ is the scattering angle). The SAXS experiments were carried out at 15 °C with a sample volume of 40 µl in a vacuum-tight quartz capillary. Radiation damage for the protein sample was monitored with six 5 min exposures, and no radiation effect was observed. The data were normalized to the intensity of the transmitted beam. The scattering of the buffer was subtracted, and the difference curves were scaled with protein concentration. All the data processing steps were performed using the program PRIMUS^68^ from the ATSAS package. The experimental data obtained for all protein samples were analyzed for aggregation using the Guinier region. The forward scattering, I(0), and the radius of gyration, *Rg*, were computed using the Guinier approximation. These parameters were also computed from the extended scattering patterns using the indirect transform package GNOM^69^, which provided the distance distribution function, *P(r)*, the maximum particle dimension, *Dmax*, and the radius of gyration, *Rg*. The qualitative particle motion was inferred by plotting the scattering patterns in the normalized Kratky plot^45^ and Porod-Debye plot^70^. *Ab initio* low-resolution models of the proteins were built by the program DAMMIN which considers low angle data (q < 2 nm^−1^)^71^. Twenty independent *ab initio* reconstructions were performed for each protein and then averaged using DAMAVER^72^. The averaged and filtered *ab initio* model was superimposed with the atomic model using SUPCOMB^73^. The theoretical scattering curves from the atomic structures were generated and evaluated against the experimental scattering curves using CRYSOL^46^. Rigid body modeling was performed using CORAL^74^ by docking the individual domains of the high-resolution structures against the experimental data. The oligomeric state of the protein was confirmed from the molecular mass calculation based on *I(0)*, Porod volume (*Vp*), excluded volume (*Vex*) and the volume of correlation (*Vc*)^75,76^.

### Modeling of Aβ40/L-PGDS complex using MD simulations

The initial structure of L-PGDS was taken from Lim *et al*., 2013 (PDB ID 4IMN)^31^. The structure of Anticalin US7 in complex with Aβ40, (PDB ID 4MVI)^47^ contained residues 16–28 of Aβ40 resolved, was used to construct the model of L-PGDS in complex with Aβ40. Since L-PGDS contains the lipocalin fold which is structurally similar to Anticalin US7, it is reasonable to assume that Aβ40 would bind in a structurally similar manner towards L-PGDS as that of Anticalin US7. The structure of the complex of Anticalin US7 with Aβ (16–28) (PDB ID 4MVI) was aligned to the structure of L-PGDS (PDB ID 4IMN), and coordinates of L-PGDS (from 4IMN) and Aβ (16–28) (from 4MVI) were extracted to build the initial model of L-PGDS with Aβ (16–28). The unresolved residues of Aβ40, i.e., segments D1-Q15 and G27 to V40, were modelled in an arbitrary extended conformation using Discovery Studio 4.1. The L-PGDS-Aβ40 complex was subjected to steepest descent energy minimization in-vacuo using the Gromacs 5.1.2 package^77^ and the charmm36M force field^78^, until a force convergence of 1000.0 kJ/mol/nm is achieved.

In order to study the binding mode of Aβ40 in complex with L-PGDS, we performed classical molecular dynamics simulations using the Gromacs 5.1.2 package. The CHARMM36m force field was used, whereby the parameter sets have been recently optimized for simulations of intrinsically disordered and folded proteins. The system was solvated with CHARMM-modified TIP3P^79^ water in a cubic box with a distance of 1.2 nm from the solute to the box edge. Na+ and Cl− counterions were added to neutralize the system and to achieve a salt concentration of 0.15 M. Bonds containing hydrogen atoms were constrained using the LINCS^80^ algorithm, to enable a time step of 2 fs. Particle Mesh Ewald^81^ was used with a cutoff of 1.2 nm for electrostatics, and a cutoff of 1.2 nm was used for van der Waals interactions. Steepest descent energy minimization was performed until a force convergence of 1000.0 kJ/mol/nm is reached to remove any initial bad contacts. An equilibration of 1 ns in the NVT ensemble was performed before the start of production simulation. The temperature of the system was maintained at 300 K using the V-rescale^82^ thermostat. 5 sets of classical molecular dynamics simulations were performed with different random initial velocities for 300 ns. Trajectory frames were saved every 2 ps.

The last 200 ns of each simulation repeat was combined to produce a 1 µs trajectory of sampling. Analysis was conducted using built-in Gromacs tools. The Aβ40 radius of gyration was computed using gmx gyrate tool. In order to identify the representative conformations of the L-PGDS- Aβ40 binding, we performed geometric clustering of the simulation frames using the Gromos algorithm. The clustering group was selected as the Aβ40 peptide, using a cutoff of 0.8 nm. Clustering was performed for every fifth frame to minimize statistical correlation.

### Extraction of aggregated proteins from human brain tissue

To check the disaggregase role of L-PGDS, we extracted aggregated proteins from post-mortem brain tissue samples of a 69-year-old male diagnosed with subarachnoid hemorrhage and dementia, which was obtained from the brain bank of the Choju Medical Institute of the Fukushimura hospital (Toyohashi, Aichi, Japan). The scientific use of human material like a brain tissue sample was conducted following the Declaration of Helsinki, and informed consent was obtained from the guardians of the patients. All procedures were approved and performed in accordance with the ethical guidelines of the Nanyang Technological University ethics board. Brain tissue was first dissected, and the large blood vessels removed. Then tissue was cut into small pieces and homogenized in the buffer comprising 2% SDS, 50 mM ammonium acetate (pH 6.0). The cell debris was removed by centrifugation at 3000 × g, 10 °C for 10 min. The supernatant was collected and ultra-centrifuged at 112000 × g, 10 °C for 2 h. The pellet was considered as aggregated proteins since these proteins were insoluble in 2% SDS and 50 mM ammonium acetate buffer (pH 6.0). Then, the pellet was washed with the buffer containing 20 mM HEPES, 100 mM NaCl, 2 mM TCEP (pH-7.5), and the supernatant was discarded. Thus, all the buffer soluble proteins were removed before treating the aggregates with WT-L-PGDS, Hexafluoroisopropanol (HFIP), and formic acid. Formic acid is a common solvent used to dissolve protein aggregates from brain and HFIP is a universal solvent used to break aggregates in β-sheet peptides. So, HFIP and formic acid treated samples acts as positive controls in this study to compare the effect of L-PGDS.

After treatment all the samples were centrifuged and only the soluble proteins in the supernatant were loaded on to SDS PAGE and separated at 100 V. The protein bands were visualized using coomassie brilliant blue. The gel lane was cut and processed for in-gel digestion after reduction with dithiothreitol (10 mM) and alkylation with iodoacetamide (55 mM) as described earlier. The gel pieces were subjected to sequencing grade modified trypsin (Promega, Madison, WI) digestion at 37 °C overnight. The tryptic peptides were extracted using 50% acetonitrile/5% acetic acid, vacuum centrifuged to dryness and subjected to LC-MS/MS analysis. LC-MS/MS analysis was performed using QExactive mass spectrometers (Thermo Fisher, MA) coupled with a Dionex RSLC nanoLC system. 5 μl sample was injected into an acclaim peptide trap column via the auto-sampler of the Dionex RSLC nanoLC system. Mobile phase A (0.1% FA in 5% acetonitrile) and mobile phase B (0.1% FA in acetonitrile) were used to establish a 60 min gradient. The peptides were analyzed on a Dionex EASY-spray column (PepMap C18, 3 μm, 100° A) using an EASY nanospray at an electrospray potential of 1.5 kV. The MS was recorded at 350–1600 *m*/*z* at resolution 70,000 *m*/*z*. Maximum accumulation time was set at 100 ms, and dynamic exclusion was 30 s. MS/MS spectra resolution was 35,000 at *m*/*z* 200; ACG was 1 × 10^6^ for the full MS and 2 × 10^5^ for the MS2 scan. For HCD, 10 most intense ions above 1000 count threshold were chosen, and 120 ms was set for maximum ion accumulation. The collision energy was 28 and an isolation width of 2 Da for the MS2 scan. Single charged and unassigned ions were excluded from MS/MS. For HCD, normalized collision energy was set to 28. The underfill ratio was defined as 0.1%.

### Data analysis

Proteome Discoverer (PD, Thermo Scientific, San Jose, USA) ver 1.4 software was used to analyze MS data acquired by QExactive. The MS/MS spectra were deisotoped and deconvoluted using the MS2 spectrum processor. Database search was conducted using a Mascot (version 2.2.06, Matrix Science, Boston, MA) search engine against an in-house built database and Uniprot human database (Released on 7/25/2016, 70,849 sequences, 23,964,784 residues). The peptide precursor mass tolerances of 10 ppm and 0.02 Da mass tolerance was used during data search. The fixed modification was carbamidomethylation (+57.021 Da) of cysteine residues, while deamidation (+0.984 Da) of asparagine and glutamine residues and oxidation (+15.995 Da) of methionine residues were variable. The resultant search output was exported to an excel file for further analysis.

## Supplementary information

Supplementary Information

Supplementary Table S1

## Data Availability

The datasets generated during and/or analyzed during the current study are available from the corresponding author on reasonable request.
