# Supplementary material for: Abundant neuroprotective chaperone Lipocalin-type prostaglandin D synthase (L-PGDS) disassembles the Amyloid-β fibrils
Source: Sci Rep. 2019 Aug 29;9:12579. doi: 10.1038/s41598-019-48819-5 (PMC6715741; doi:10.1038/s41598-019-48819-5)
Supplement: Supplementary file 1 — Supplementary Information [file 41598_2019_48819_MOESM1_ESM.pdf]

Supplementary Information

Abundant neuroprotective chaperone Lipocalin-type prostaglandin D synthase (L-PGDS) disassembles the Amyloid-β fibrils

Bhuvaneswari Kannaian[1], Bhargy Sharma[1], Margaret Phillips[1], Anup Chowdhury[1], Malathy S S Manimekalai[1], Sunil S Adav[1,2], Justin T Y Ng [1], Ambrish Kumar[3], Sierin Lim[3], Yuguang Mu[1], Siu K Sze[1], Gerhard Grüber[1] and Konstantin Pervushin[1]*

[1] School of Biological Sciences, Nanyang Technological University, Singapore-637551.

[2] Singapore Phenome Centre, Lee Kong Chian School of Medicine, Nanyang Technological University, Singapore, 636921

[3] School of Chemical and Biomedical Engineering, Nanyang Technological University, Singapore-637459.

*Correspondence to kpervushin@ntu.edu.sg

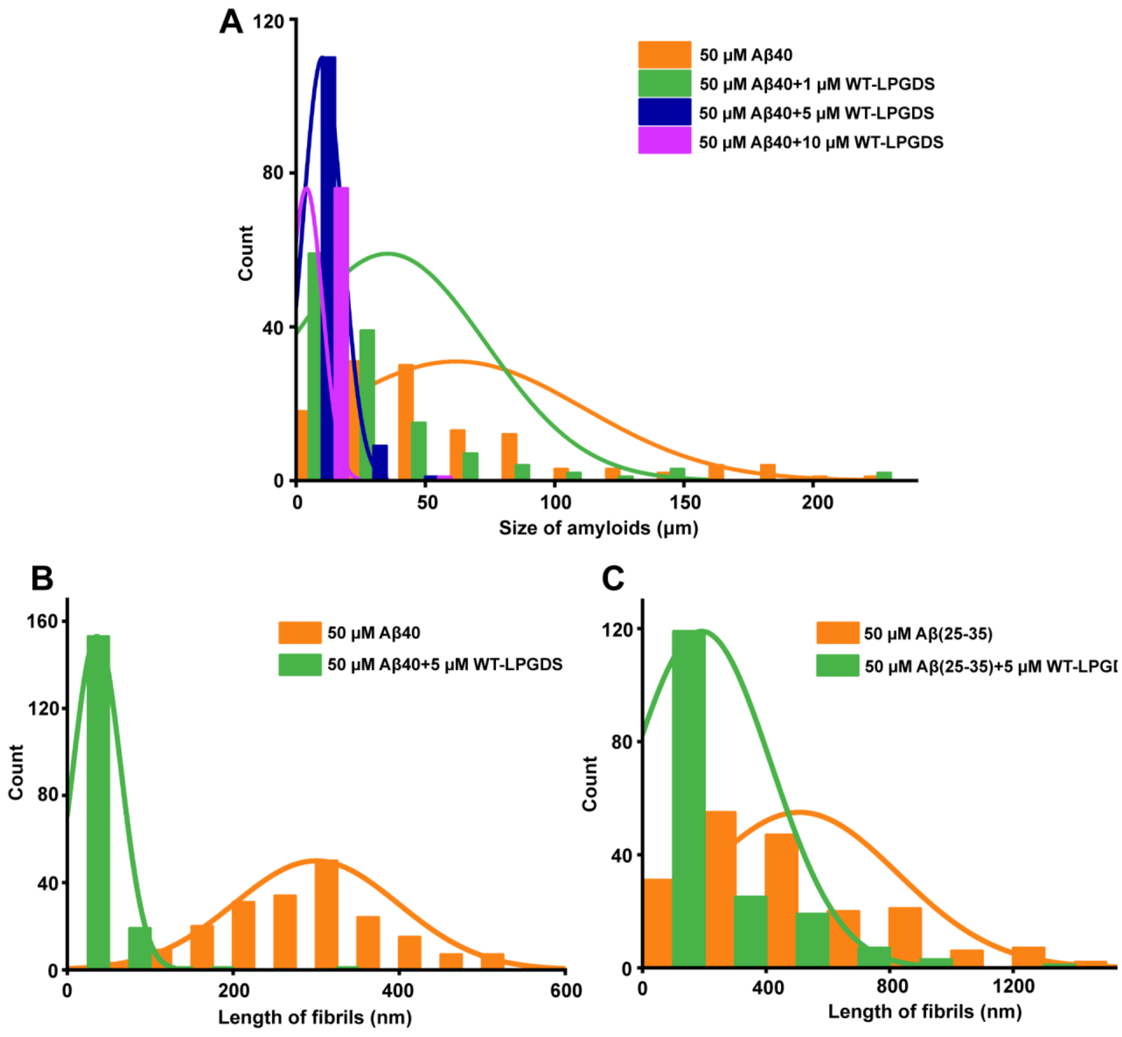

S1: (A) Quantitative analysis of amyloids formed for 50 µM Aβ40 (orange) incubated with 1 µM (green), 5 µM (blue) and 10 µM WT- L-PGDS (magenta) calculated using fluorescence microscopy images (B,C) distribution on fibril length for 50 µM Aβ40 and Aβ(25-35) (orange) and after disintegration by 5 µM WT- L-PGDS (green) calculated using transmission electron microscopy images.

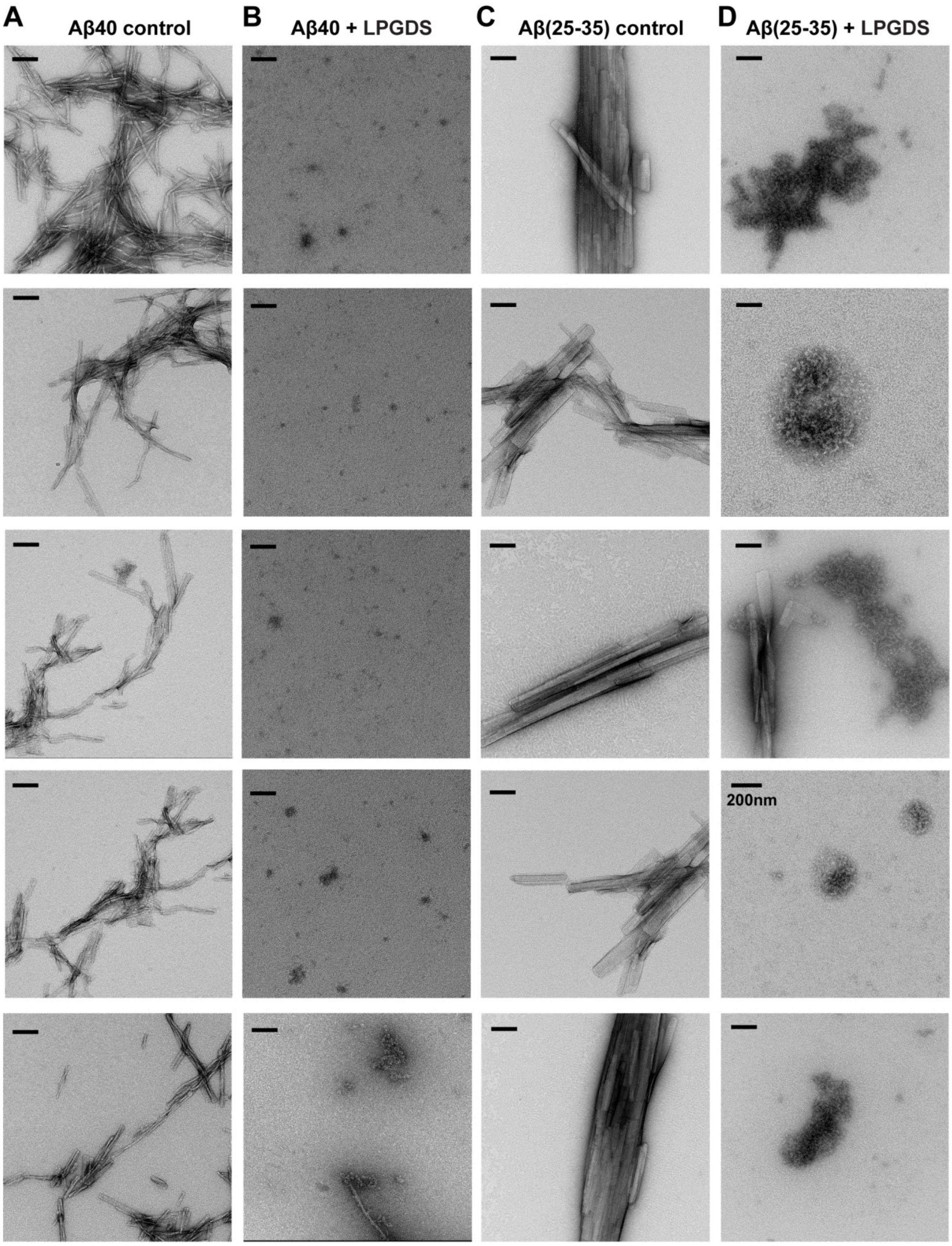

S2: Representative TEM images for (A) Aβ40 control (B) Aβ40 fibrils treated with 5 μM WT-L-PGDS (C) Aβ(25-35) control (B) Aβ(25-35) fibrils treated with 5 μM WT-L-PGDS. (Scale bar: 100nm).

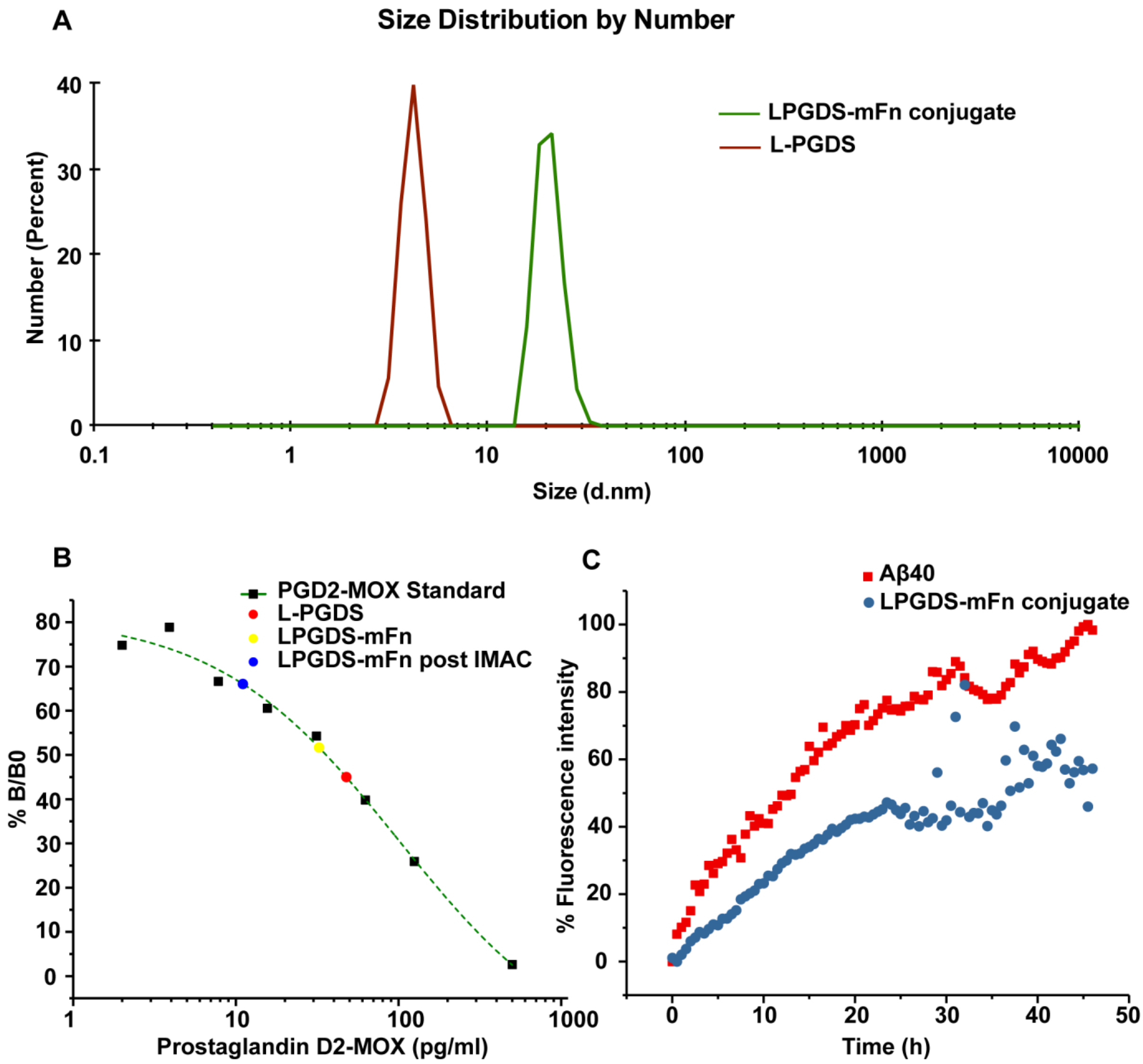

S3: (A) Difference in size of L-PGDS-ferritin conjugate shown by Dynamic Light scattering (DLS). Size distribution by number shows that peak at 4nm for L-PGDS is shifted to 20nm in case of the conjugate. Data acquired in Malvern Zetasizer Nano series equipment (B) Functionality test for L-PGDS-ferritin conjugates using PGD2-MOX ELISA kit (Cayman Chemicals) calculated by plotting absorbance values on standard curve using a four-parameter curve fit. Y-axis represents percent ratio of sample absorbance to maximum absorbance. X-axis implies amount of PGD2-MOX prepared in pg/ml. For 500 pg/ml initial concentration of PGH2 added in L-PGDS or conjugate samples approx. 47 pg/ml, 32 pg/ml, and 11pg/ml PGD2-MOX was produced by L-PGDS only, L-PGDS-ferritin conjugate before IMAC, and L-PGDS-ferritin conjugate after IMAC, respectively. Data acquired on Cytation plate reader, analyzed using Originlab (C) Thioflavin fluorescence assay shows inhibition of Aβ40 fibril formation (initial concentration 50 μM) by 5μM L-PGDS-ferritin conjugate. The intensity for sample with L-PGDS-ferritin conjugate added to Aβ40, reduced to 50% as compared to only Aβ40 control after 48 hours of incubation at 37°C. Data acquired on cytation plate reader, analysed using Originlab.

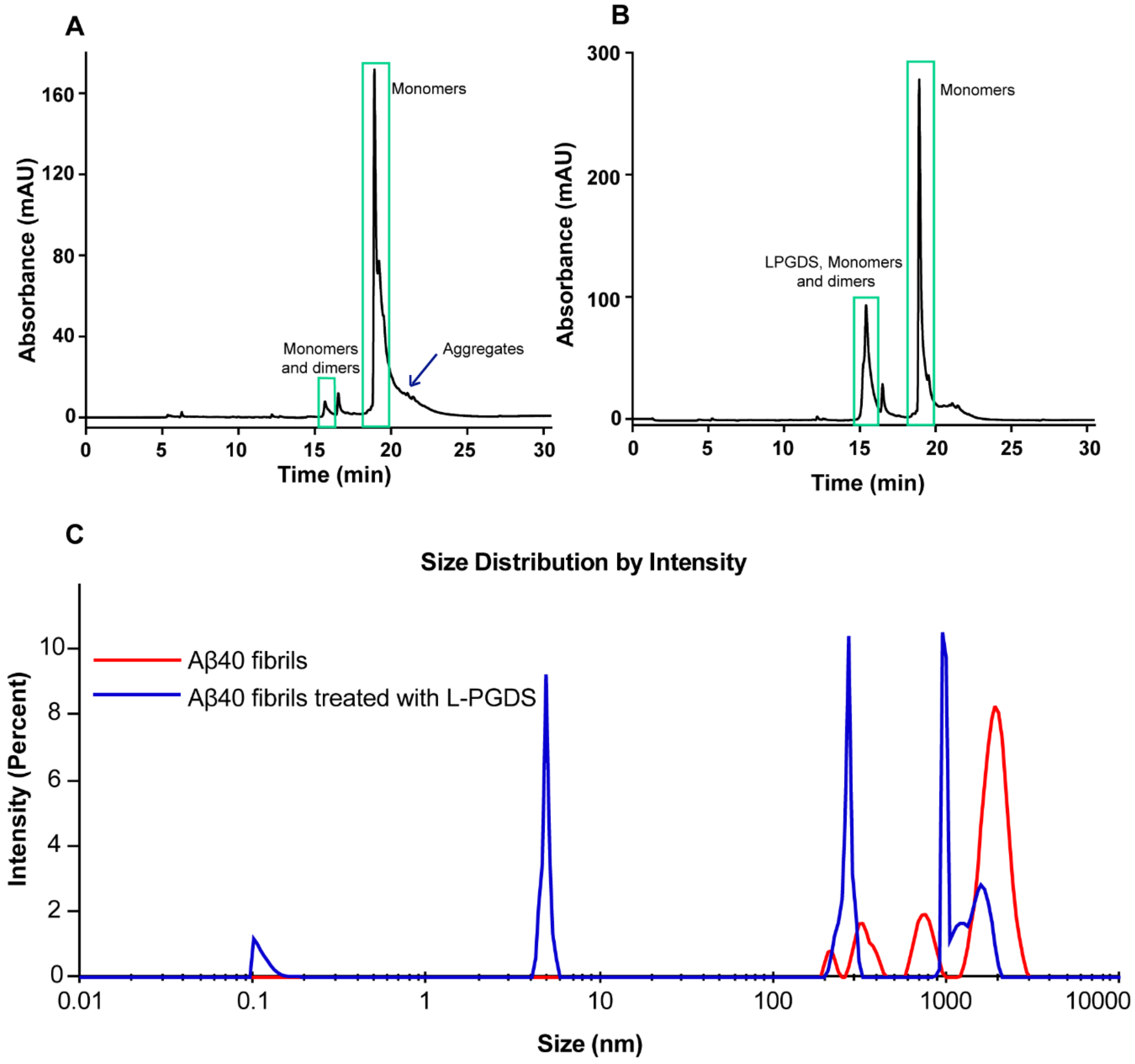

S4: (A, B) HPLC chromatogram for Aβ40 fibrils and Aβ40 fibrils treated with WT-L-PGDS respectively. (C) Overlay of size distribution by intensity for untreated Aβ40 fibrils (red) and Aβ40 fibrils treated with L-PGDS (blue). The peak at 4nm corresponds to L-PGDS.

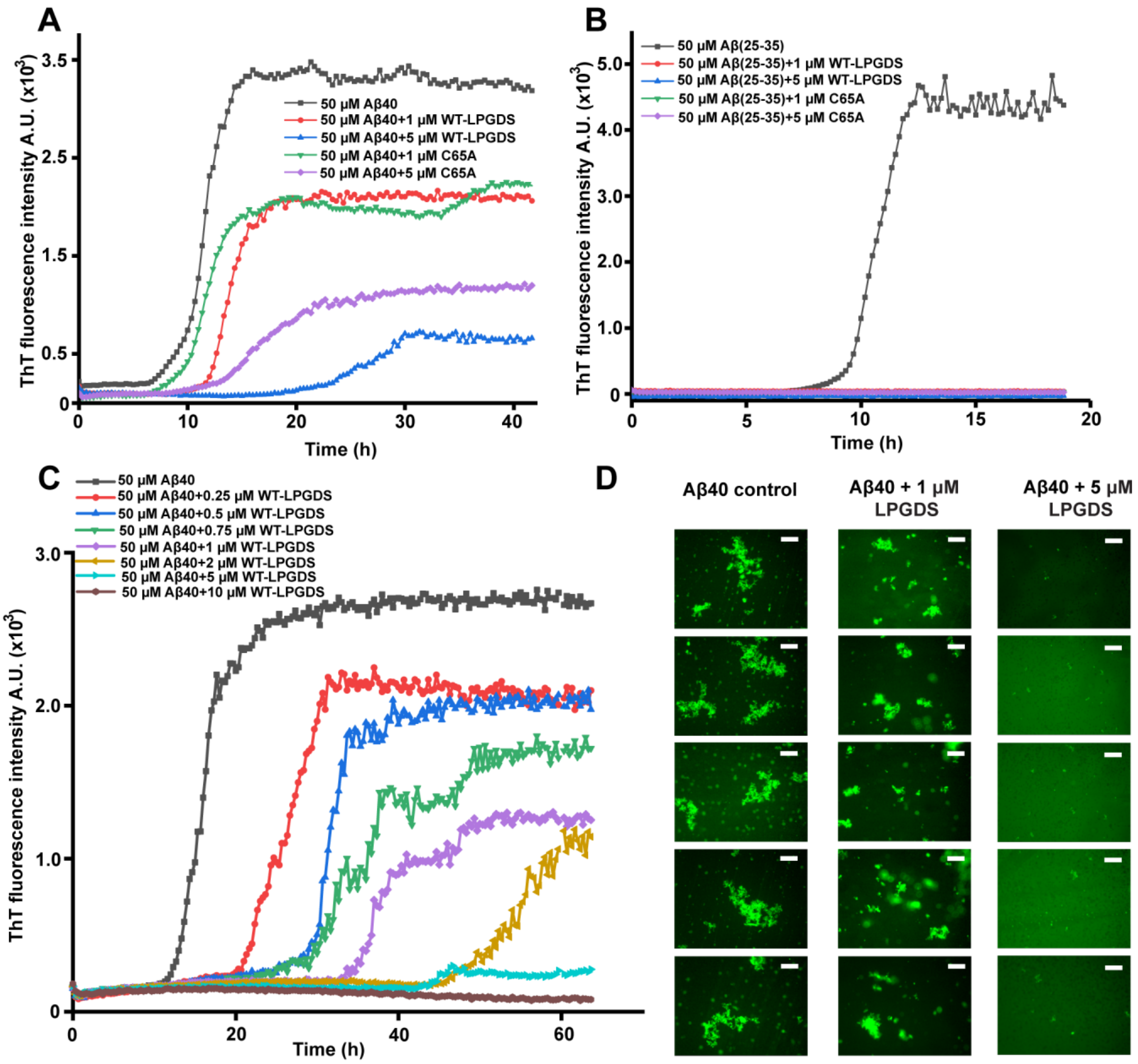

S5: (A, B) ThT curve for 50 µM Aβ40 and Aβ(25-35) (black) incubated with 1 µM (red) and 5 µM WT- L-PGDS (blue) or 1 µM (green) and 5 µM C65A mutant of L-PGDS (magenta) (C) ThT fluorescence plot for 50 µM Aβ40 incubated with increasing concentrations of WT-L-PGDS. This data has been used for IC50 calculation (D) Representative fluorescence images for Aβ40 control and Aβ40 incubated with 1 µM and 5 µM WT-L-PGDS.

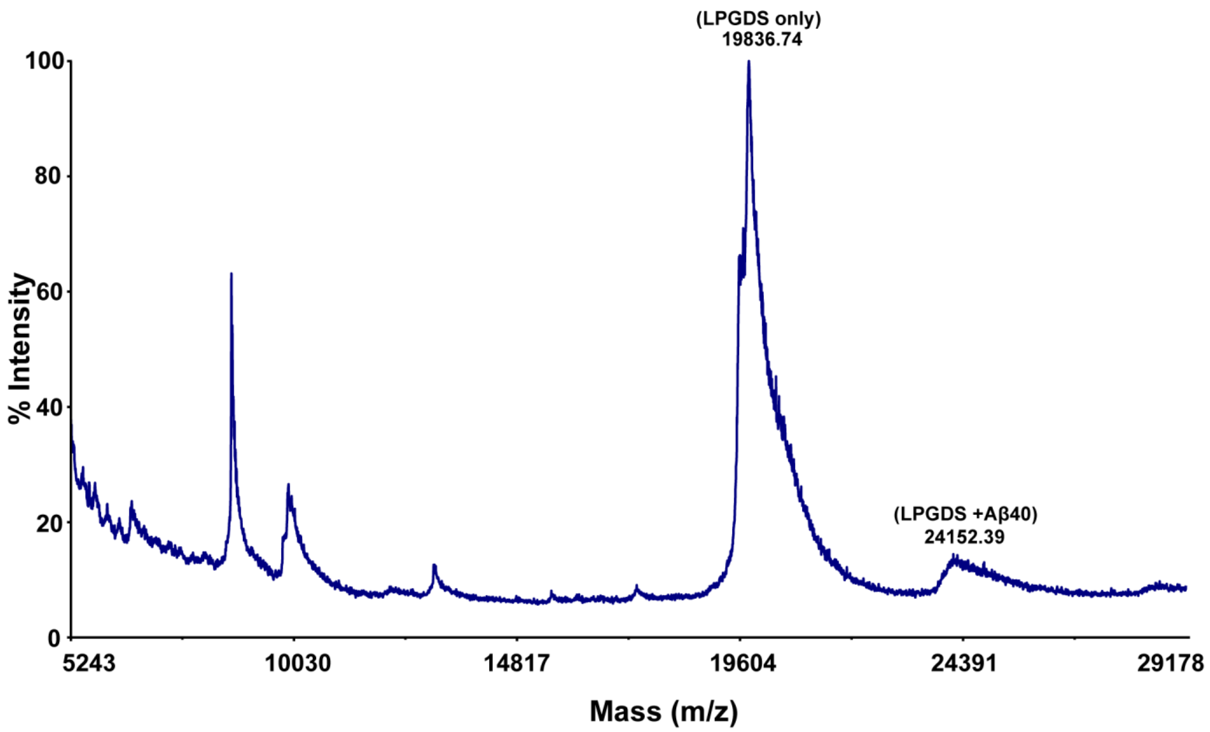

S6: MALDI-TOF MS spectrum of L-PGDS in a complex with Aβ40. The peak at 19.8 kDa represents L-PGDS and the peak at 24.1 kDa represents the 1:1 complex of L-PGDS-Aβ40 (19.8 kDa + 4.3 kDa). No peak for 1:2 (L-PGDS:Aβ40) complex is detected.

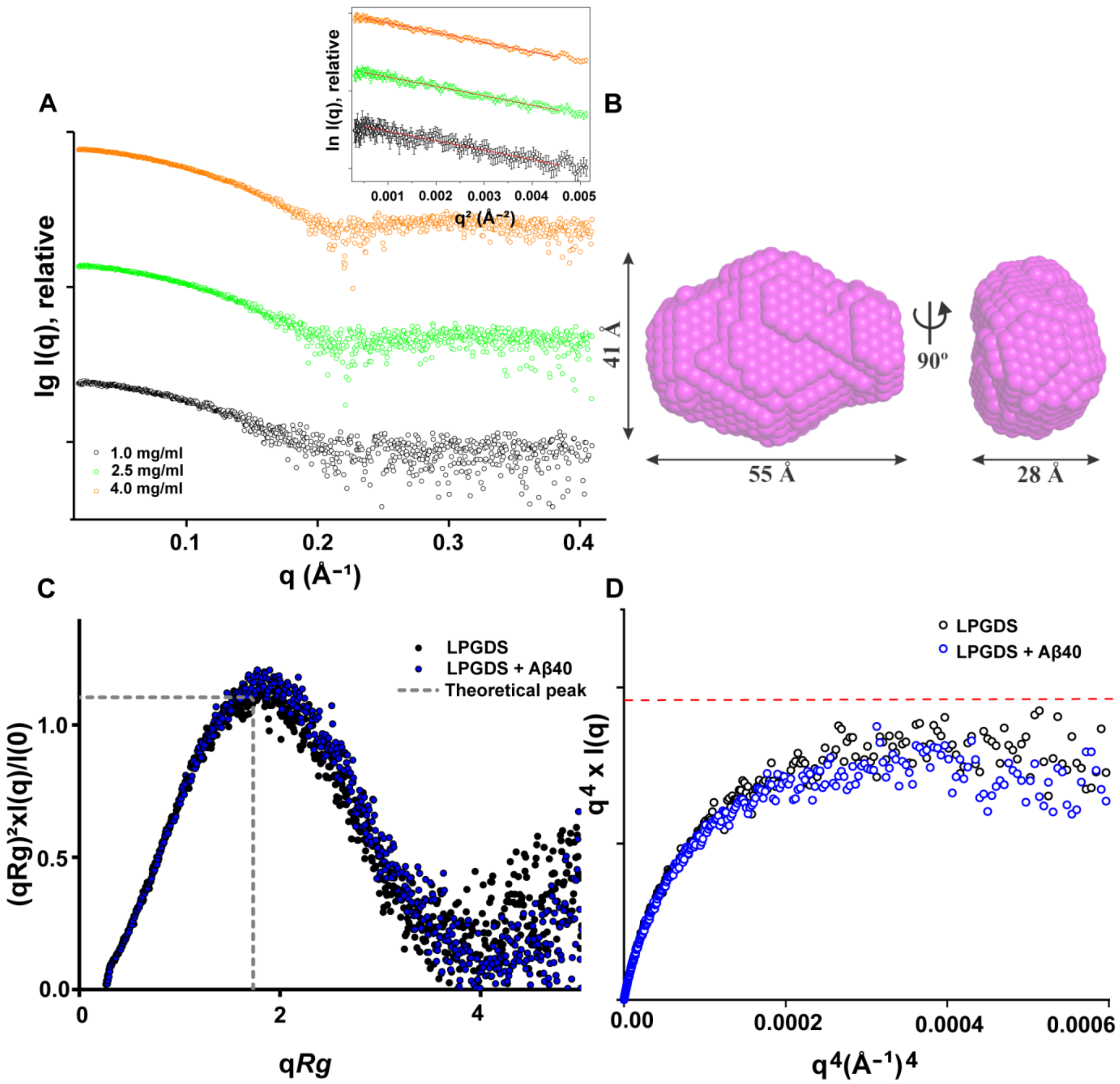

S7: (A) Solution X-ray scattering pattern (○) of L-PGDS at 1.0 mg/ml (black), 2.5 mg/ml (green) and 4.0 mg/ml (orange) concentration. (Inset) Guinier plots show linearity at all concentrations used, indicating no aggregation. The scattering profiles are offset for clarity by applying arbitrary scale factors. (B) The averaged and filtered envelope of L-PGDS with bilirubin (magenta) from ten independent ab initio reconstructions using DAMMIN show similar shape as that of L-PGDS alone. (C) Normalized Kratky plot of L-PGDS (●; black) compared to L-PGDS and Aβ40 (●; blue) with a peak (---; grey), representing the theoretical peak and assuming an ideal Guinier region of a globular particle. (D) Porod-Debye plot of L-PGDS (○; *black*) and L-PGDS with Aβ40 (○; *blue*). The presence of Porod-Debye plateau in the plot suggests both proteins are compact in solution.

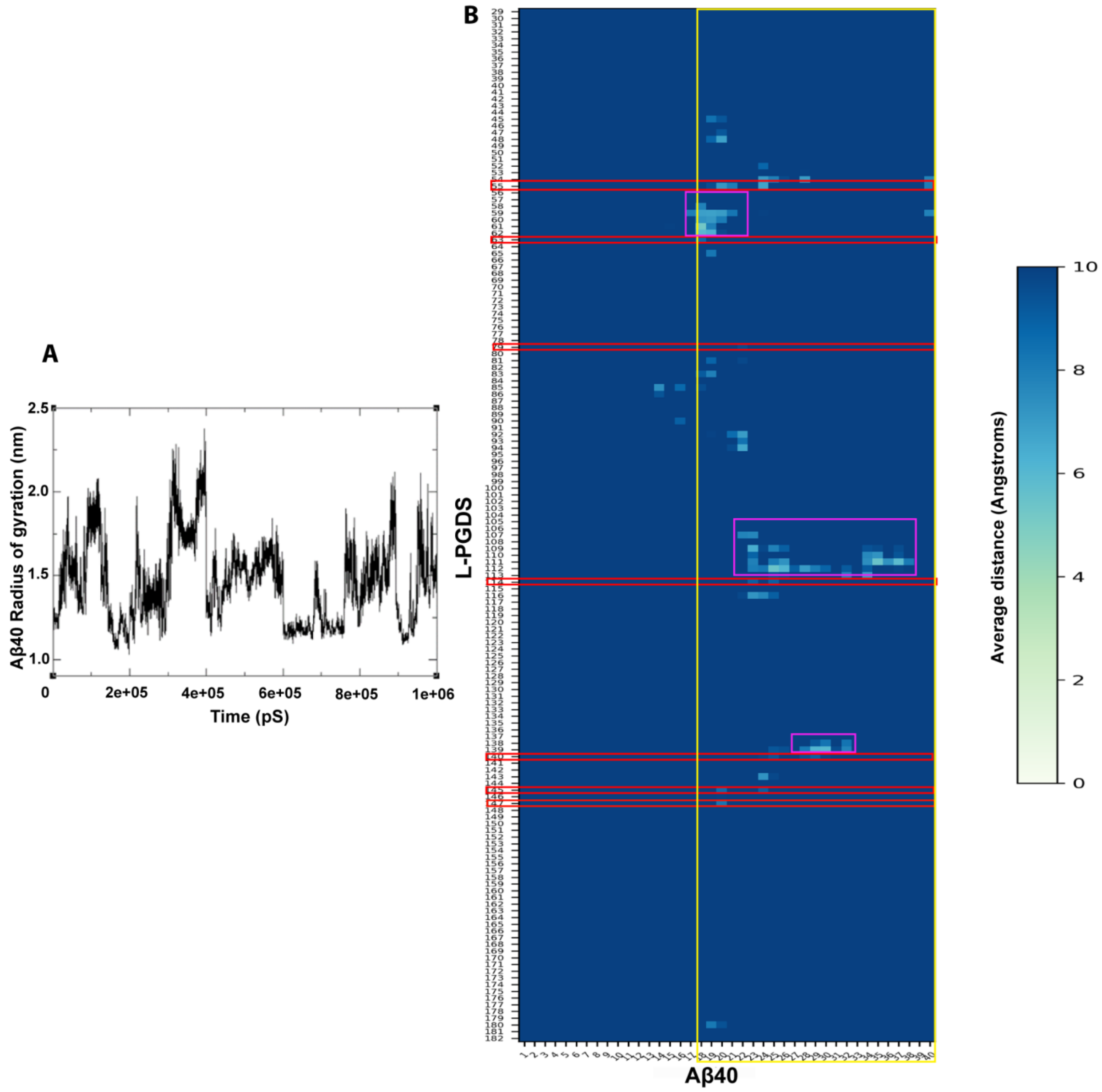

S8: (A) Radius of gyration plot for Aβ40. 5 simulations of 300ns with random initial velocities were performed for the L-PGDS-Aβ40 complex. The last 200ns of each simulation repeat was extracted and combined for radius of gyration calculation. (B) Contact map of average heavy atom residue distances between L-PGDS and Aβ40, with a range from 0 (white) to beyond 10 Å (dark blue). Distances were calculated using the Gromacs gmx distance tool over simulation frames belonging to the cluster for which the representative model is derived. NMR CSP identified L-PGDS residues are highlighted in red, while NMR CSP identified Aβ40 residues were highlighted in yellow. For clarity purposes, CSP identified residues of L-PGDS with average simulation calculated distances of more than 10 Å towards all Aβ40 residues are not highlighted, and vice versa. Simulation model identified additional contact sites between L-PGDS and Aβ40 are highlighted in Magenta.

Table. S1: Proteomic analysis of proteins solubilized from protein aggregates of AD brain tissue by L-PGDS, Formic acid and HFIP treatment. (See file: Supplementary info Table S1).

**Data collection parameters**		
Instrument (source & detector)	Bruker NanoStar equipped with MetalJet eXcillum X-ray source and VÅNTEC-2000 detector	
Beam geometry	100 μm slit	
Wavelength (Å)	0.134	
q range ($Å^{-1}$)	0.016 – 0.4	
Exposure time (min)	30 (6 frames x 5 min)	
Temperature (K)	288.15	
Protein sample	**LPGDS**	**LPGDS + Aβ40**
Concentration range (mg ml^{-1})	2.5	2.5
**Structural parameters**		
I(0) (arbitrary units) [from P(r)]	32.03 ± 0.27	38.76 ± 0.34
R_g (Å) [from P(r)]	18.04 ± 0.17	19.13 ± 0.22
I(0) (arbitrary units) (from Guinier)	32.09 ± 0.37	38.82 ± 0.38
R_g (Å) (from Guinier)	17.91 ± 0.32	18.91 ± 0.29
D_{max} (Å)	57 ± 5	65 ± 5
Porod volume estimate (V_p) ($Å^3$)	~23000	~25900
DAMMIN excluded volume (V_{ex}) ($Å^3$)	~32452	~34251
Dry volume from sequence ($Å^3$) ‡	~22710	~27928
**Molecular mass determination**		
Calculated monomeric *MM* (kDa) [from sequence*]	~19	~23
MM from *Porod invariant* (V_p/1.6) (kDa)	14 ± 2	16 ± 2
MM from *excluded volume* (V_{ex}/2) (kDa)	16 ± 2	17 ± 2
MM from *volume of correlation* (V_c) (kDa)	20 ± 2	22 ± 2
**Software employed**		
Primary data reduction	BRUKER SAS	
Data processing	PRIMUS	
Ab initio analysis	DAMMIN	
Validation and averaging	DAMAVER	
Computation of model intensities	CRYSOL	
Rigid body modeling	CORAL	
3D graphics representations	PyMOL	

* http://web.expasy.org/compute_pi/

‡ http://www.basic.northwestern.edu/biotools/proteincalc.html

Table. S2: Data collection and scattering derived parameters for L-PGDS and L-PGDS-Aβ40 complex.

Protein	Inhibit primary nucleation	Inhibit secondary nucleation	Inhibit Fibril elongation	Dis-aggregate bundle of fibres	Delay fibril growth	ATP dependency	Part of a large complex	RNA expression levels in Brain (TPM)	Reference	Location	Cerebral cortex	Hippocampus	Caudate	Cerebellum
L-PGDS	Y	Y	N	Y	Y	N	N	1224.7	Our study	intracellular, secreted	high in neuronal cells, high in endothelial cells	medium in glial cells, low in neuronal cells	low in neuronal cells, low in glial cells	medium in cells in molecular layer, low in cells in granular layer, low in purkinje cells
Clusterin (ApoJ)	Y	Y	Y	Y	Y	Y	Y	10875.4	{Narayan, 2011 }	intracellular, secreted	medium in neutrophils	medium in neuronal cells	not detected	not detected
αB-crystallin (sHsp)	Y	Y	Y	-	-	Y	Y	1888.7	{Raman, 2005;Mainz A, 2016 }	intracellular, membrane	high in glial cells, high in neutrophils	high in glial cells	high in glial cells	medium in cells in granular layer
Hsp60	Y	Y	Y	-	Y	Y	Y	141.6	{Mangione, 2016}	intracellular	low in neuronal cells, low in glial cells	low in neuronal cells, low in glial cells	low in neuronal cells, low in glial cells	low in purkinje cells
Hsp 70/40	Y	Y	Y	N	Y	Y	Y	5	{Evans, 2006}	intracellular	low in neuronal cells, low in endothelial cells, low in glial cells	low in neuronal cells, low in glial cells	low in neuronal cells, low in glial cells	low in cells in molecular layer, low in cells in granular layer, low in purkinje cells
Hsp 90-α	Y	N	Y	N	-	Y	Y	1021.5	{Evans, 2006}	intracellular secreted	low in neuronal cells	not detected	not detected	low in purkinje cells
Pyruvate kinase	Y	-	-	N	Y	Y	N	749	{Luo, 2014}	intracellular	medium in neuronal cells, medium in neutrophils	low in neuronal cells, low in glial cells	medium in neuronal cells	medium in cells in molecular layer, low in cells in granular layer
Transferrin	N	Y	Y	-	-	N	N	385.9	{Raditsis, 2013}	intracellular secreted	low in neutrophils	not detected	low in neuronal cells	not detected
Transthyretin	Y	Y	Y	-	Y	N	Y	6.9	{Li, 2013 }	secreted	not detected	not detected	not detected	not detected
α2-Macroglobulin	Y	-	N	N	Y	N	N	172	{Hughes, 1998}	secreted	low in neuronal, high in endothelial cells	low in neuronal cells	not detected	low in purkinje cells
Catalase	N	-	-	Y	Y	N	Y	47.2	{Luo, 2014}	intracellular	not detected	not detected	not detected	not detected
Lysozyme	N	Y	-	N	Y	N	N	22.5	{Luo, 2013}	secreted	not detected	not detected	not detected	not detected
Brichos domain containing proteins	N	Y	N	-	Y	N	N	7	{Cohen, 2015; Landreh, 2015 }	intracellular, membrane	not detected	not detected	not detected	not detected
Haptoglobin	Y	-	N	N	Y	N	N	1.8	{Yerbury, 2009}	intracellular secreted	not detected	not detected	not detected	not detected
Serum Albumin	N	Y	Y	-	Y	N	N	1	{Stanyon, 2012 #1337}	intracellular secreted	low in endothelial cells	low in glial cells	not detected	low in molecular layer
Apolipoprotein A1	-	Y	Y	-	-	N	N	0.1	{Paula-Lima, 2009#299}	intracellular secreted	not detected	not detected	not detected	not detected

Table. S3: Comparison of chaperone activity for traditional chaperones and non-chaperone proteins that affect amyloid- β aggregation. RNA expression levels are Courtesy of Human Protein Atlas, www.proteinatlas.org, (Uhlén M et al, Science 2015). (Y) - Yes, (N) - No, (-) Not known.
